# Monitoring Toxicity Associated with Parenteral Sodium Stibogluconate in the Day-Case Management of Returned Travellers with New World Cutaneous Leishmaniasi

**DOI:** 10.1371/journal.pntd.0001688

**Published:** 2012-06-26

**Authors:** Emily S. Wise, Margaret S. Armstrong, Julie Watson, Diana N. Lockwood

**Affiliations:** 1 Hospital for Tropical Diseases, University College London Hospitals NHS Trust, London, United Kingdom; 2 Department of Parasitology, Hospital for Tropical Diseases, University College London Hospitals NHS Trust, London, United Kingdom; Hospital Universitário, Brazil

## Abstract

**Background:**

Patients with New World cutaneous leishmaniasis (NWCL) caused by *Leishmania Viannia* are treated with parenteral sodium stibogluconate (SbV) to reduce the risk of development of mucocutanous leishmaniasis. Our centre manages patients with NWCL on an outpatient-basis. This study was conducted to assess the safety and efficacy of this approach.

**Methodology:**

We reviewed records of 67 consecutive NWCL patients, aged 17–61 years, treated as day-cases with 20 mg/kg/day SbV for up to 28 days at our UK centre. Data had been collected in a standardised format at the time of treatment using a care-record tool. Patients reported adverse-effects daily using a structured questionnaire. Blood tests and electrocardiograms were performed twice weekly to monitor for toxicity.

**Principal Findings:**

Parenteral SbV treatment was associated with an early, significant suppression of mean lymphocyte and platelet counts. By day four of treatment, lymphocytes reduced by 0.53×10^9^/L (CI 0.29×10^9^/L to 0.76×10^9^/L, *p*<0.001), and platelets by 31,000/µL (CI 16,000/µL to 46,000/µL, *p*<0.001). SbV was further associated with significant elevation of serum alanine transaminase concentrations, with a mean peak rise of 107 iu/L by day 13 (CI 52 iu/L to 161 iu/L, *p*<0.001). These disturbances were temporary and did not result in adverse clinical events. Patient-described symptoms were cumulative and at three weeks of treatment, 59.6% of patients experienced myalgia and 29.8% malaise. Treatment adherence and clinical outcomes were comparable to inpatient treatment studies. A total of 1407 individual doses of SbV resulted in only 26 nights' hospital admission, a saving of 1381 bed-days compared to inpatient treatment.

**Conclusions/Significance:**

In specialist centres, NWCL patients aged below 65 years and without co-morbidities can be safely and effectively treated without hospital admission. This reduces the cost of treatment, and is much preferred by patients. Twice weekly blood and electrocardiographic monitoring may be surplus to requirement in clinically well, low-risk patients.

## Author Summary

Sodium stibogluconate (SbV), a pentavalent antimonial, administered parenterally, is the recommended treatment for South American cutaneous leishmaniasis, caused by *Leishmania Viannia*, which is a neglected disease that affects many people resident in Central and South America, as well as travellers to the areas. Antimonials have been used for the treatment of leishmaniasis since the 1930s. We report the toxicity experienced by a series of NWCL patients receiving SbV in a resource-rich setting. This study also evaluates administration of the drug to patients without admitting them to hospital. The administration of parenteral SbV was associated with myelosuppression, derangement of markers of liver function and prolongation of the QT interval on electrocardiography, although these effects were not found to be associated with adverse clinical events, and the majority of doses of SbV were administered without cause for hospital admission. Our data shows that parenteral SbV treatment may be provided with reduced monitoring for toxicity than is currently done, and on an outpatient-basis, without endangering safety. Such practice, with reduced demands on local finances and the healthcare workforce, would be desirable in more resource-limited settings.

## Introduction

Cutaneous leishmaniasis (CL) develops after inoculation of the skin with protozoan parasites of the genus *Leishmania*, transmitted by the bite of phlebotomine sandflies. Cutaneous nodules form and then ulcerate, typically within weeks of initial infection. The annual worldwide incidence of CL is estimated at 1–1.5 million cases, with over 90% of cases occurring in the Middle East and South America [1]. CL may be classified by the geographical origin of the parasite, and New World cutaneous leishmaniasis (NWCL) describes CL endemic to Central and South America, most frequently caused by *L.viannia* and *L.mexicana* complex. Infection with *L.viannia* can be complicated by mucocutanoeus leishmaniasis (MCL), when parasites spread to the nasopharyngeal mucosa and produce local destruction [2]. Among indigenous people in rural Bolivia, untreated CL was estimated to progress to MCL in an estimated 5–20% of patients [3]. At the Hospital for Tropical Diseases, London, UK (HTD) between 1995 and 2003 we saw 81 cases of active or healed *L.viannia* NWCL, six of which were complicated by MCL [4]. MCL does not heal spontaneously, is disfiguring and can be fatal. It is important to identify the subgenus of the infecting organism of a NWCL lesion, as parenteral treatment is required in all cases of *L.viannia* to reduce the risk of developing MCL. Parenteral therapy is also recommended when it is not possible to identify the NWCL species or in ‘complicated’ *L.mexicana* complex infections (where there are multiple or large lesions, lesions involve the face or a joint, or there is involvement of the lymphatic system), to aid primary lesion healing [5]. Non-complicated, non-*L.viannia* lesions (both from the New World and Old World) should self-heal without dissemination. However, parenteral, intra-muscular or intra-lesional pentavalent antimonials are still recommended as first line treatment in this instance, to accelerate cure and to reduce scarring [2].

The World Health Organisation and the Centres for Disease Control recommend a course of 20 mg/kg/day of sodium stibogluconate (SbV) for 20 days to treat NWCL caused by *L.viannia* [6]–[12]. The administration of SbV is associated with transient toxicities, which have been previously characterized. However, many of the reports on SbV toxicity were published more than 20 years ago or have limitations. In 2011, Oliveira *et al* published the first systematic review on the adverse effects of treatments for NWCL and approved only 65 studies on pentavalent antimonials for NWCL as suitable for inclusion, with a total of only 937 patients treated in clinical trials. Furthermore, disparity between treatment regimens employed by different centres, the variability in the antimonium content in each batch of drug produced and a paucity of quantifiable data meant few firm conclusions could be drawn [13].

Patient-described side effects are cumulative and dose-related. A 1998 study of 96 military personnel with NWCL treated with the standard regime reported mylagia in 55%, fatigue in 39%, abdominal pain in 29% and nausea and vomiting in 27% [14].

SbV administration is associated with bone marrow suppression. Hepburn described transient thrombocytopaenia complicating a case of NWCL after standard SbV treatment [15]. Wortmann *et al* published a prospective study in 1997 of eight NWCL patients in which the white cell and lymphocyte counts were demonstrated to have decreased on day seven of standard SbV treatment [16]. There are case reports of cutaneous and meningitic varicella zoster virus infection during treatment, which have been attributed to SbV-induced leucopenia [16]–[18].

Standard SbV treatment for NWCL is known to derange liver function. Lawn *et al* demonstrated that in 65 patients, 85% developed a rise in serum hepatic transaminases concentrations. Levels declined after two weeks, despite ongoing treatment [19]. In a study of six patients, Hepburn *et al* demonstrated acute hepatocellular damage and a fall in the functional metabolic capacity of the liver (demonstrated by a reduction in caffeine clearance) that was rapidly reversible on stopping treatment [20]. There are no published reports of fulminant hepatic failure secondary to SbV treatment for NWCL. Elevated serum amylase levels are very commonly associated with SbV administration. Gasser *et al* showed 48 out of 49 patients receiving standard SbV had elevated amylase levels [21]. However, clinical pancreatitis is rare and severe or fatal pancreatitis extremely rare [22].

Electrocardiogram changes have been described to occur in 50% of patients receiving standard parenteral SbV treatment for NWCL, although cardiac toxicity with tachyarrhythmias is most likely limited to patients with pre-existing cardiovascular pathology [13], [19], [23]–[25].

Serious adverse events due to SbV are rare, and deaths are very rare. When serious events do occur, they are most often due to cardiac arrhythmias or pancreatitis.

Further data on the toxicity of SbV are available from clinical trials where it has been used to treat visceral leishmaniasis (VL) in African countries. Higher rates of toxicity are seen with higher doses of parenteral SbV. Bryceson *et al* attempted to treat ten cases of VL in Kenya with 60 mg/kg/day of SbV, administered in three divided doses, but this regimen was modified or abandoned in six of the cases due to suspected toxicity [26]. However, it is inappropriate to compare treatment data for patients with systemic VL in Africa and patients with cutaneous-limited disease in the UK.

In NWCL-endemic countries, centres may both be familiar with managing the condition and have limited resources and therefore parenteral SbV treatment is usually ambulatory, with hospitalisation often only restricted to a small percentage of patients with co-morbidities or experiencing complications [13]. This is contrary to many centres treating imported NWCL in non-endemic, resource-rich settings, where many clinicians follow the recommendations of Davidson, who advocated inpatient treatment, and monitoring with twice-weekly blood tests and electrocardiograms. Davidson recommended suspending treatment for 1–2 days when liver enzymes reached more than ten times the normal level, amylase three times the normal level, the QTc interval is prolonged to greater than 500 ms or in the event of a cardiac arrhythmia. Davidson's recommendations were based on the limited amount of data available on SbV toxicity and were therefore not strictly evidence-based, giving arbitrary thresholds for treatment suspension [23].

In contrast, in the 2003 study, Lawn *et al* found SbV toxicity to be of little clinical significance [19]. Therefore since 2003, treatment for NWCL at HTD is now provided in an outpatient setting, provided that the patient is in good general health, less than 65 years old, and with no known renal, hepatic or cardiac disease. Outpatient care was standardised with the development of a protocol-driven Integrated Care Pathway (ICP). A copy of the HTD ICP can be found in the Supporting Information (Protocol S1). A prospectively maintained patient database of patients with NWCL has been kept at our centre since the introduction of the ICP, and it is from this database that this study has been conducted. Since 2003, the HTD has treated up to 20 cases a year of NWCL with parenteral SbV. Cases are identified either when patients self-present to the HTD returned travellers' walk-in clinic or are referred to our centre by general practices, dermatology clinics and military hospitals from across the UK.

This retrospective survey had three aims. The first was to determine the toxicity of SbV, when administered at 20 mg/kg/day for 3–4 weeks. This was done both by examining liver, renal and bone marrow function blood tests taken throughout the course of treatment, and by examining the experienced effects prospectively reported by the patients in a daily structured questionnaire. Patients were asked at every treatment if they had symptoms of nausea, myalgia, skin rashes, abdominal pain and/or malaise. The second aim of this study was to determine adherence to the treatment protocol outlined in the ICP by both patients and staff. The ICP recording tool was examined to see whether patients observed the requirement to attend daily for drug administration. The third aim was to determine the efficacy of delivering treatment on an outpatient basis in British patients. There may be differences in responses to treatment and experienced effects by different ethnic group. Prior to the current report, outpatient treatment with parentral SbV in the UK has only been reported in a study on 13 marines [17].

## Methods

### Ethics Statement

Our institutional review board (IRB) assessed the project and ruled that this project was a retrospective review and a clinical audit and exempted the author group from requiring formal approval. All patient data analysed was anonymised, with patient-identifying information removed, obviating the need for patient consent.

### Case Definition

Patients were either diagnosed with NWCL after presenting to the HTD walk-in clinic, or were referred to our centre by another hospital or general practitioner. Lesions indicative of NWCL were biopsied for microscopy with Romanovsky type-staining (Rapid Field's), culture with modified Novy-McNeal-Nicolle medium and polymerase chain reaction (PCR) for leishmania DNA. Specimens were also examined histologically.

A case of NWCL requiring parenteral SbV was defined as a typical lesion(s) in a returned traveler from an endemic country in Latin America with confirmation of leishmania infection in at least one laboratory modality and either identification of *L.viannia* DNA on PCR or identification of a ‘complex’ case with multiple or large lesions or lesions involving the face, a joint, or the lymphatic system. Patients were offered outpatient treatment on the ICP if they meet the following criteria: under 65 years of age, able to attend daily; no pre-existing cardiac or renal condition; normal full blood count, urea and electrolytes and liver function tests; and normal electrocardiogram with corrected QT interval less than 421 milliseconds.

We identified all patients who received a course of parenteral SbV for NWCL on the ICP at our centre from April 2003 (when the ICP was introduced) until September 2008 from a prospectively maintained clinical research database.

### Description of Management of NWCL on the ICP

Patients travelled to the hospital daily from home or a local hotel. Treatment was for 21 days, which was extended up to 28 days, at the discretion of the attending consultant, if lesion healing was unsatisfactory. Treatment was administered, the lesion was assessed and adverse effects were documented, in accordance with the ICP protocol (summarised in Table 1, complete copy of the ICP in Supporting Information (Protocol S1)), which was printed in a booklet that also served as a data-recording tool. The nursing staff reviewed and dressed each lesion daily, supplemented by a more detailed weekly inspection. The dose of SbV (Pentostam®, Wellcome, Brentford, Middlesex, UK) was 20 mg/kg, with no upper limit, diluted in 100 ml of 0.9% saline and administered over 30 minutes via a peripheral venous cannula that was changed every third day. Temperature, pulse and respiratory rates, and blood pressure were measured before, during and after treatment. Nursing staff documented daily if the patient was experiencing myalgia, a skin rash, nausea, malaise or abdominal pain. The team duty doctor was informed if there was any adverse event. Each patient was also medically reviewed every third day, and a full blood count, serum electrolytes, liver function tests and a 12-lead electrocardiogram obtained. Serum amylase was only checked if pancreatitis was clinically suspected. Interruption of treatment was at the discretion of the attending consultant.

**Table 1:** The ‘Integrated Care Pathway (ICP)’ framework for patient monitoring.

Day of treatment assessment performed	Assessment or intervention	Member of staff performing assessment
Pre-treatment, 1, 4, 7, 10, 13, 16, 19, 21, (24), (28)	Full blood count, urea and electrolytes, liver function tests, electrocardiogram	Doctor or nurse, results reviewed by doctor
Daily	Lesion condition and dressing	Nurse
1, 7, 13, 21, (28)	Lesion location, size, grade, floor condition, surrounding skin condition, exudate, odour, pain, infection status and plan for lesion dressing	Nurse
Daily	Screening questionnaire for patient-experienced effects	Nurse
Daily	Blood pressure, temperature, respiratory rate and heart rate before, during and after treatment	Nurse
Pre-treatment, 1, 4, 7, 10, 13, 16, 19, 21, (24), (28)	Patient review	Doctor

See Supporting Information (Protocol S1) for a complete copy of the ICP record-of-care proforma.

### Data Collection and Statistics

A retrospective analysis was performed on the case notes, the ICP case recording tool and laboratory results for each patient. The reference ranges of the hospital's laboratories were utilised as the normal ranges for serum concentrations of haemoglobin (11.5–15.5 g/dL), lymphocytes (1.20–3.65×10^9^/L), eosinophils (0.0–0.4×10^9^/L), platelets (150,000–400,000/µL), creatinine (49–92 µmol/L), bilirubin (6–23 µmol/L), and alanine transaminase (10–35 iu/L). Patients' symptoms during treatment had been recorded prospectively, using the daily screening questionnaire. The case notes were examined for clinical events suggestive of disordered haemostasis, immune suppression or liver failure. We examined the ICP for the incidence of prolongation of the QTc (over 420 ms), as recorded by the clinicians reviewing the ECGs at time of treatment. The ECGs themselves were not analysed in this study, as this aspect of toxicity has been reported in previous studies [19], [24], [25].

Repeated measures analyses-of-variance (ANOVAs) was performed on the haematological and biochemical laboratory values measured prior to initiation of treatment and on days four, seven, 10, 13, 16, 19 and 21 of treatment. Pairwise differences were calculated for combinations for each of the dates (multiple comparison corrected (Bonferroni) analysis) and p-values were calculated. The level of the tests for significance was 0.05. Data were analysed by SPSS for windows version 16.0 (SPSS Inc., IBM, Somers, New York, USA).

## Results

### Clinical Features of Patients

Sixty-seven patients (50 male) with NWCL were treated using the ICP between April 2003 and September 2008. The mean age was 30 years (range 17–61 years). None of the patients had human immunodeficiency virus infection (routine screening was not performed), and none had a significant co-morbid disease. Belize was the most common country of acquisition (32/67, 47.7%) and 25/67 patients were serving in the British Armed Forces and had acquired their CL during military training in the jungles of Belize. Other countries of acquisition were Peru (7/67), Bolivia (7/67), Guyana (5/67), Costa Rica (4/67), Brazil (3/67), Ecuador (3/67), Mexico (2/67), Guatemala (1/67) and Columbia (1/67). Of the 67 patients, 51 had infection with *L.viannia*, 12 had infection with *L.mexicana* complex, in one patient insufficient protozoal DNA was obtained to determine the infecting species, in two patients PCR was not performed and in one no DNA was found. Systemic therapy was given to the patients with *L.mexicana* complex infections as there were facial lesions (4/12), large or deep lesions (4/12), multiple lesions (3/12) or extensive lymphadenopathy (1/12). One third of all patients treated (24/67) had more than one cutaneous lesion. Lesions were most common on exposed areas (distal to the elbows, distal to the knees, or over the head and neck (85/112 of the total number of lesions). There was sporotrichoid spread or lymphadenopathy in lymph nodes adjacent to the skin lesions, palpable on clinical examination, in 19/67 of the cases. No patients had clinical evidence of mucocutaneous infection at the time of treatment. Two of the 67 patients had received prior treatment for their NWCL: one patient had received 21 days of intramuscular SbV while in Argentina, the second had received parenteral SbV, suspended after 12 doses due to deranged liver function, and amphotericin, stopped due to an allergic reaction, at another UK hospital.

### Adherence to Treatment

Table 2 documents the number of daily treatments the patients received. 61/67 (91.0%) received at least 20 days treatment. All causes for interruptions to treatment are documented in Table 3. 12/67 patients (17.9%) had disrupted treatment due to toxicity or experienced effects. Patient adherence to the treatment protocol was high, with only two patients failing to attend for six days in total. Only one treatment was omitted due to nursing error. However, in five patients in whom treatments were suspended, staff failed to compensate with additional treatment days at the end of expected treatment.

**Table 2:** Total number of SbV doses received by each of the 67 patients.

Total number of treatment doses administered	Number (%) of patients
9	1/67 (1.5)
12	1/67 (1.5)
13	1/67 (1.5)
16	1/67 (1.5)
19	2/67 (3.0)
20	5/67 (7.5)
21	43/67 (64.2)
22	3/67 (4.5)
24	1/67 (1.5)
25	1/67 (1.5)
28	7/67 (10.4)
Insufficient data	1/67 (1.5)

**Table 3:** Reasons for treatment suspensions and omissions.

Patient	Reason for drug omission	No. of days treatment was suspended
A	Platelet count dropped to 14×10^9^/L	7
B	Abdominal pain, nausea, myalgia	1
C	Urticarial rash	2
D	Vomiting and abdominal pain	1
E	Fevers, rigors and headache	4
F	Fevers and unwell	2
G	Patient did not attend	5
H	Nursing error	1
I	Neutrophil count dropped to 0.83×10^9^/L	4
J	Patient did not attend	1
K	QTc prolonged to 524 ms	1
L	Patient developed a MSSA bacteraemia	Treatment terminated day 14
M	ALT elevated at 447 iu/L	10
N	ALT elevated at 492 iu/L	5
O	Developed chest pain during treatment	Half of dose omitted

### Treatment Response

Additional intra-lesional SbV treatment, to assist primary lesion healing, was administered to two patients while they underwent the ICP, and to a further five patients at outpatient follow-up. One of these patients was also given a course of allopurinol to aid healing. These patients are considered presumptive parenteral SbV treatment failures. 19/67 (28.4%) patients failed to attend a follow-up appointment following the completion of treatment on the ICP, often, in the case of military personnel, due to redeployment abroad. Of the 48 patients not lost to follow-up, 29 (60.4%) and 38 (79.2%) patients had lesions that had completely re-epithelialised within two and six months of completing treatment with parenteral SbV respectively (and had not required additional intra-lesional treatment). No patients have reported with re-occurrence of cutaneous disease. Only one of the 44 cases infected with *L.viannia* developed MCL - 16 months after completing the ICP, the DNA of *L.viannia* was detected following a nasal biopsy to investigate persistent nasal stuffiness.

### Treatment Toxicity

The mean weight of the female patients was 62 kg and mean daily dose of SbV given was 1230 mg (range 1000 mg–1600 mg). The mean weight for male patients was 78 kg and mean daily dose of SbV given was 1560 mg (range 1300 mg–1800 mg). The study did not identify an obvious association between patients receiving a larger dose of SbV per day (31/67 patients received over 1500 mg/SbV/day) and the toxicity experienced.

**Figure 1:**
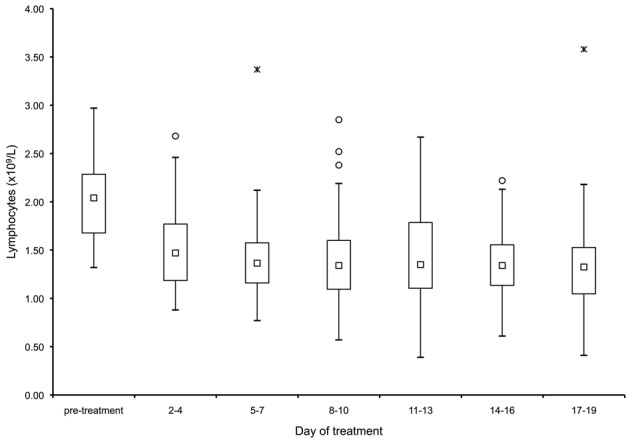
Box-and-whisker plot of the lymphocyte counts of patients plotted against duration of treatment with sodium stibogluconate (SbV). The group median (squares) fell significantly from its baseline by the first subsequent measurement, four days after initiating treatment (*p*<0.001). It then plateaued, with no further significant change. Outliers are depicted by circles, extremes are depicted by hashes.

There was a significant drop in lymphocyte counts between pre-treatment levels and all subsequent lymphocyte levels throughout the course of treatment (Figure 1). The median lymphocyte count prior to treatment was 2.06×10^9^/L, falling to 1.57×10^9^/L four days after initiating treatment, a reduction of 0.53×10^9^/L, confidence Intervals (CI) 0.29×10^9^/L to 0.76×10^9^/L, *p*<0.001 (repeated measures ANOVA). There was no further significant change in the lymphocyte count, between days seven and 19, after the initial reduction. Although the median lymphocyte count never dropped below the lower end of normal reference range of 1.2×10^9^/L. There were no documented events of opportunistic infections in our patient group. NWCL was not associated with an eosinophilia (mean eosinophil count pre-treatment 0.25×10^9^/L), nor did the eosinophil count change during treatment.

**Figure 2:**
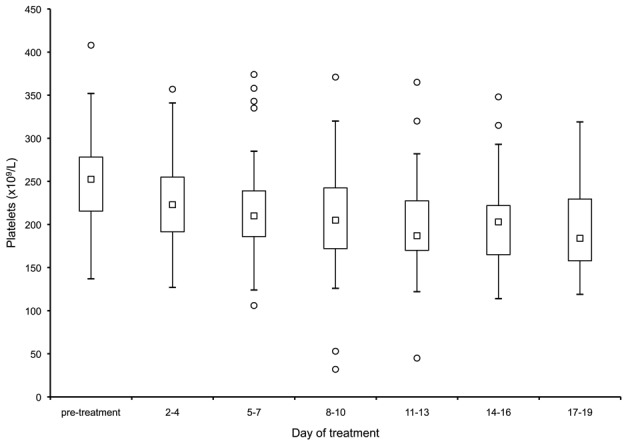
Box-and-whisker plot of the platelet counts of patients plotted against duration of treatment with sodium stibogluconate (SbV). The group median (squares) fell significantly from its baseline by the first subsequent measurement, four days after initiating treatment (*p*<0.001). It then plateaued, with no further significant change. Outliers are depicted by circles, extremes are depicted by hashes.

There was a small but significant drop in the median platelet count in response to treatment (Figure 2). The median platelet count decreased from 252,000/µL before the initiation of treatment to 222,000/µL on the fourth day (a fall of 31,000/µL, CI 16,000/µL to 46,000/µL, *p*<0.001). There was no further significant fall in the platelet counts between days seven to 19. Throughout treatment, the median platelet count never dropped below the lower end of normal reference range of 150,000/µL, and there were no documented events of bleeding. However, two patients became thrombocytopaenic, (with platelet counts of 32,000/µL and 54,000/µL respectively), both after ten days of treatment. There were no bleeding events, treatment was suspended for seven days in the former patient and continued in the latter, and in both the count recovered to within the normal reference range. There was a small fall in mean haemoglobin concentration by day 19 (1.0 g/L, CI 0.59 to 1.43, *p*<0.001).

**Figure 3:**
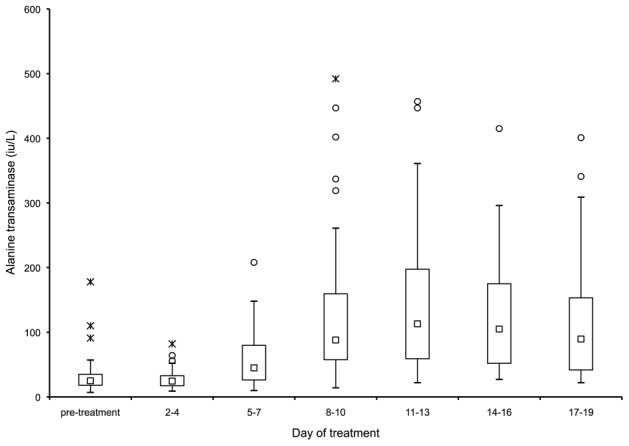
Box-and-whisker plot of serum alanine transaminase concentrations against duration of treatment with sodium stibogluconate (SbV). The group median (squares) rose significantly by day 10 after initiation of treatment (*p*<0.001). Outliers are depicted by circles, extremes are depicted by hashes.

Serum alanine transaminase (ALT) concentrations rose significantly after starting treatment, with considerable individual variability (Figure 3). The peak median rise was 107 iu/L by day 13 (CI 52 iu/L to 161 iu/L, *p*<0.001). The levels decreased thereafter, despite ongoing treatment, though this was not significant. In one patient, the ALT rose to 492 iu/L by day 10, and treatment was suspended for five days. The level had declined to 278 iu/L by day 19. None of the patients developed a clinically apparent hepatitis. Serum bilirubin and creatinine levels did not rise during treatment. Serum amylase levels were not routinely checked in the patient group. No patient in the group was clinically suspected of having pancreatitis. The peak incidence for reported abdominal pain was on day 18 of treatment (in five of the 55 patients questioned), some of which may have represented symptomatic hyperamylasemia.

QTc prolongation (range 422–524msec) occurred in nine out of 67 patients, on days four to 19 of treatment. There were no documented events of clinically suspected tachyarrhythmias.

**Figure 4:**
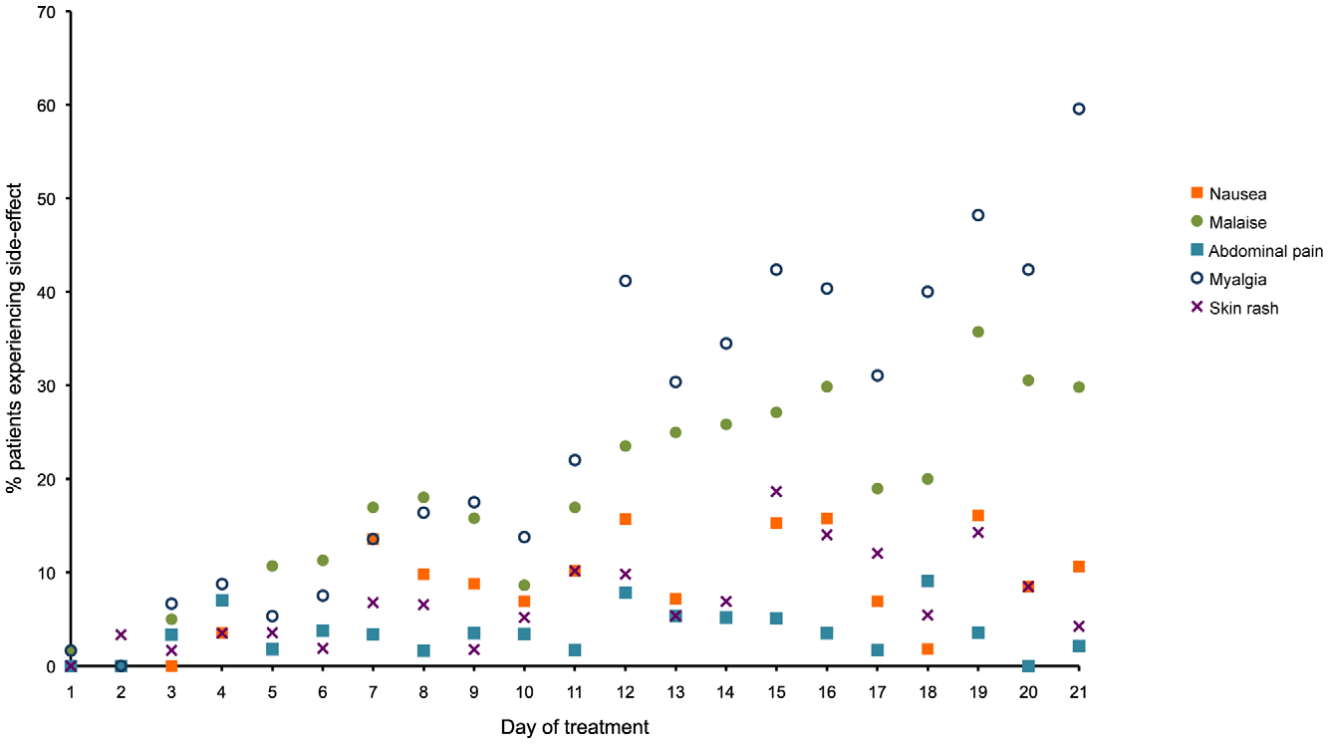
Percentage of patients reporting side-effects on each day of treatment with sodium stibogluconate (SbV).

The responses to the symptom questionnaire are summarised in Figure 4. Out of a total of 66 patients for whom there was sufficient data, 63 (95.5%) reported at least one adverse reaction. The timing for peak incidences for side effects was: day 15 for skin rashes (11/59 of patients whose response was recorded), day 18 for abdominal pain (5/55 patients), day 19 for nausea (9/56 patients), day 19 for general malaise (20/56 patients) and day 21 for myalgia (28/47 patients).

Although myalgia was common, no patients were suspected to have rhabdomyolysis. Creatine kinase was not routinely checked. For most patients these side effects were mild and transient, and resolved soon after completion of treatment. One patient, who had a history of eczema, had worsening eczema during treatment, which persisted for six months. One patient developed pelvic pain that persisted for four months after treatment finished, but no pelvic pathology was found on investigation.

3/67 patients required one night's hospital admission for drug-related side effects (one for myalgia, one for vomiting, and one for pyrexia). This was out of a total of 1407 doses of drug administered. One patient had their treatment terminated on day 14 for a methicillin-sensitive *Staphylococcus aureus* bacteraemia secondary to an infected cannula site, which responded to intravenous antibiotics. One patient was admitted for three nights within a week of completing treatment with abdominal pain, vomiting, headache and a temperature that settled with supportive treatment. One patient was admitted for six nights during his treatment as he was of no fixed abode. In total, for the 67 patients treated on the ICP, 1381 bed days were saved.

## Discussion

This study has demonstrated that NWCL can be managed successfully on an outpatient basis, with good clinical outcome, patient adherence to the treatment regime, and staff adherence to the ICP protocol. The majority of doses were administered without complication, and overall treatment was well tolerated without major toxicity. However, almost all patients experienced the adverse symptoms associated with a course of parenteral sodium stibogluconate. The use of an outpatient regime was preferred by patients and represents a major financial saving.

Although this was a retrospective study, the standardised ICP protocol enhanced the quality and quantity of relevant data that could be surveyed and analysed. The rigorous laboratory monitoring of patients on SbV demonstrated that there were falls in the lymphocyte, platelet and haemoglobin counts during the course of treatment, but that this haematological suppression was not clinically significant, despite a steep drop in platelet counts in two patients. It is not possible to predict which patients will experience thrombocytopaenia with SbV: we found no relationship between the initial platelet count of individuals receiving treatment and subsequent decreases in platelet count. Serum alanine transaminase became elevated, but in a transient and predictable manner, and without detectable adverse effect. Monitoring of bilirubin levels and renal function was demonstrated to be unnecessary. Repeated electrocardiography on patients undergoing treatment did demonstrate transient QTc prolongation in some patients, but in this cohort this did not result in clinically apparent cardiac arrhythmias. We cannot exclude the possibility that some patients experienced silent arrhythmias that might have become apparent with prolonged recordings.

We propose that, at appropriate treatment centres, all patients with NWCL under the age of 65 years, able to attend daily, with no pre-existing cardiac or renal condition and with a normal full blood count, serum electrolyte and liver function profile and an electrocardiogram with a normal QTc interval, be considered for day-case treatment with a treatment protocol. Our data shows that not all patients require twice-weekly biochemical and electrocardiographic monitoring: in healthy low-risk individuals, asymptomatic lymphopaenia, thrombocytopaenia, hepatic transaminitis, hyperamylasaemia and QTc prolongation may well be of no clinical significance and resolve despite continuation of treatment. It could be argued that monitoring for these toxic effects sometimes engendered anxiety, in both the clinician and the patient, and may have resulted in unnecessary temporary suspension of treatment. This may have delayed eradication of infection in these patients, thereby increasing the time to healing and the risk of lesion reoccurrence and parasite dissemination. We propose that monitoring in asymptomatic patients should be limited to only a repeat full blood count, liver function tests amylase on day 10 of SbV-treatment, where the clinician may consider suspension of treatment for a maximum of seven days if the platelet count is less than 50×10^9^/L or the ALT is ten times or more the upper limit of normal. Monitoring with electrocardiograms of young otherwise healthy individuals with no history of heart disease is not necessary if the antimony dose does not exceed 20 mg/kg/day, as previously recommended by Chulay and colleages [25].

During a course of parenteral SbV, the emphasis should be on symptomatic relief for patient-described toxicity (most patients experienced at least myalgia by the third week of treatment). Before starting a course of SbV patients should be advised that they are likely to experience at least one of nausea, myalgia, malaise, a skin rash and/or abdominal pain and should be reassured that these symptoms are, on the whole, benign and transient. There is anecdotal evidence that administering glucocorticoids in patients receiving parenteral SbV may provide symptomatic relief, although there is insufficient evidence to justify co-administration of glucocorticoids with SbV, unless it is being used in the management of urticaria [27]. Future studies on SbV should be directed towards optimising both pharmacological and non-pharmacological management of patient's symptoms.

Management of NWCL as an outpatient may ease the burden of treatment to staff, patients and health economies. Local hotel accommodation was arranged if daily commuting from home was not feasible for the patients. This remained a cheaper option than hospital admission.

SbV is an effective drug against *L.viannia* CL, and for now is likely to remain the mainstay of its treatment in United Kingdom treatment centres. However, despite our recommendations that the use of SbV in NWCL does not require as close monitoring as previously felt necessary, it remains a drug with appreciable transient and mild side effects. Furthermore, the development of a novel treatment for NWCL that could be administered orally rather than parenterally and/or for a shorter period than the current three weeks would be preferable and better suited for more resource-poor settings than the UK. Miltefosine is an oral preparation and is currently under evaluation for the treatment of NWCL. It is emerging as a treatment option for CL in South America, where rates of lesion healing achieved with SbV are lower than in UK centres. Unfortunately, the side-effect profile of miltefosine in not insignificant and adverse effects occur with a similar frequency in miltefosine administration as to SbV, albeit of a different nature, with vomiting, nausea and diarrhoea most commonly observed. Two recent clinical trials, each of 60 patients receiving miltefosine, reported no serious adverse events [28], [29]. The need remains for new drug development for one of the world's most neglected diseases.

### Limitations

This case series is a retrospective review and so may be less rigorous than a prospective study, although the use of the ‘Integrated Care Pathway’ protocol in the patients' management and its recording tool has enhanced the quality of our data. As would be expected in any retrospective review, some data was inadequate and in particular we had only limited data on time to lesion healing. The reporting of side effects was subject to bias from both the patient and healthcare provider and ideally the efficacy and toxicity of a drug should be studied in a double-blind randomized control trial as opposed to an observational study. Although our case series of 67 NWCL patients is the largest produced by a UK centre, it remains a relatively small study.

## Supporting Information

Protocol S1 **The Hospital for Tropical Diseases, London, Integrated Care Pathway (ICP) case-record tool for the treatment of day case cutaneous leishmaniasis.**
(DOC)
