# Correction: Monitoring Toxicity Associated with Parenteral Sodium Stibogluconate in the Day-Case Management of Returned Travellers with New World Cutaneous Leishmaniasis

**DOI:** 10.1371/annotation/15ab4f39-31e7-40bc-9b15-6e90a612b49a

**Published:** 2012-09-26

**Authors:** Emily S. Wise, Margaret S. Armstrong, Julie Watson, Diana N. Lockwood

The word "Leishmaniasis" is misspelled in the title. The correct title is: Monitoring Toxicity Associated with Parenteral Sodium Stibogluconate in the Day-Case Management of Returned Travellers with New World Cutaneous Leishmaniasis. The correct citation is: Wise ES, Armstrong MS, Watson J, Lockwood DN (2012) Monitoring Toxicity Associated with Parenteral Sodium Stibogluconate in the Day-Case Management of Returned Travellers with New World Cutaneous Leishmaniasis. PLoS Negl Trop Dis 6(6): e1688. doi:10.1371/journal.pntd.0001688

