# Supplementary material for: Monitoring Toxicity Associated with Parenteral Sodium Stibogluconate in the Day-Case Management of Returned Travellers with New World Cutaneous Leishmaniasi
Source: PLoS Negl Trop Dis. 2012 Jun 26;6(6):e1688. doi: 10.1371/journal.pntd.0001688 (PMC3383730; doi:10.1371/journal.pntd.0001688)
Supplement: Protocol S1 — The Hospital for Tropical Diseases, London, Integrated Care Pathway (ICP) case-record tool for the treatment of day case cutaneous leishmaniasis. (DOC) [file pntd.0001688.s001.doc]

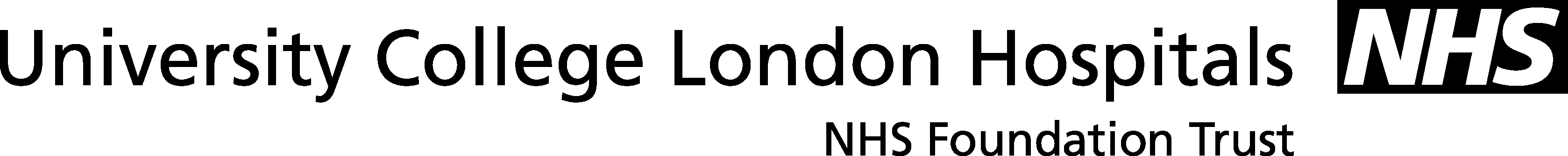


# INTEGRATED CARE PATHWAY

| **Treatment of Day Case**  **Cutaneous Leishmaniasis** Hospital for Tropical Diseases,Floor 8, University College Hospital Version 4 (October 2006) |
| --- |

# Confidential Patient Information

| **Patient Name**  **(or attach ID Label)** |  |
| --- | --- |
| **Hospital Number** |  |
| **Date of Birth** |  |
| **Male / Female** |  |
| **Address** | **Tel (Home):** |
|  |  |
|  | **Tel (Work):** |
|  |  |
|  | **Mobile:** |
|  |  |
| **Hotel Stay Patient**  **Room number:** | **Y/N** |
| **General Practitioner** |  |
| **Assessment Date** |  |
| **Date left Tropics** |  |
| **Countries Visited** | Date |
| **Length of stay** |  |
| **Purpose of Travel** |  |

| **Allergies and potential adverse incidents to drugs (please write clearly)** |
| --- |

**ICP Review Date: October 2007**

| Day Case - Cut Leishmaniasis ICP | AFFIX PATIENT ID LABEL HERE | Date |  |
| --- | --- | --- | --- |

| **Acceptable abbreviations for use in this pathway** | | | | |
| --- | --- | --- | --- | --- |
| **S/N** | **Staff Nurse** |  | **P** | **Pulse** |
| **HTD** | **Hospital for Tropical Diseases** |  | **T** | **Temperature** |
| **ICP** | **Integrated Care Pathway** |  | **BP** | **Blood Pressure** |
| **N/A** | **Not applicable** |  | **RR** | **Respiratory Rate** |
| **IV** | **Intravenous** |  | **FBC** | **Full Blood Count** |
| **OPA** | **Out Patient Appointment** |  | **U&E** | **Urea and Electrolytes** |
| **ECG** | **Electrocardiogram** |  | **LFT** | **Liver Function Test** |
| **O2Sats** | **Oxygen saturation** |  |  |  |
| **Dip / Cert Trop. Nurs.** | |  | **Diploma /Certificate in Tropical Nursing** | |

| **ALL STAFF SIGNING FOR CARE IN THIS ICP MUST RECORD BELOW DETAILS OF THEIR FULL NAME, POSITION AND SAMPLE SIGNATURE** |
| --- |

| **FULL NAME –**  **Please Print** | **POSITION** | **SIGNATURE** | **INITIAL** | **Contact**  **Number** |
| --- | --- | --- | --- | --- |
|  |  |  |  |  |
|  |  |  |  |  |
|  |  |  |  |  |
|  |  |  |  |  |
|  |  |  |  |  |
|  |  |  |  |  |
|  |  |  |  |  |
|  |  |  |  |  |
|  |  |  |  |  |
|  |  |  |  |  |
|  |  |  |  |  |
|  |  |  |  |  |
|  |  |  |  |  |
|  |  |  |  |  |
|  |  |  |  |  |
|  |  |  |  |  |
|  |  |  |  |  |
|  |  |  |  |  |
|  |  |  |  |  |
|  |  |  |  |  |
|  |  |  |  |  |
|  |  |  |  |  |
|  |  |  |  |  |

| Day Case - Cut Leishmaniasis ICP | AFFIX PATIENT ID LABEL HERE | Date |  |
| --- | --- | --- | --- |
| **INFORMATION ABOUT THIS INTEGRATED CARE PATHWAY (ICP)**  **What is an ICP?**  It is a multidisciplinary patient record developed using the best evidence to support the predicted pathway of the patient’s care.  It aims to ensure that the care we offer is of the highest quality using the best evidence in an efficient way.  **Who developed this pathway?**  **Staff nurse Veronica Barrett-hall , with assistance from Wayne**  **How do I use this pathway?**  This ICP is a guideline of the best-expected multidisciplinary care for a patient. However, remember that every patient is an individual. This ICP is NOT a substitute for your clinical judgement and expertise.   - This ICP begins with the diagnosis of cutaneous leishmaniasis at the outpatients clinic. Guidance and recording of care are according to this ICP from the time that the result of laboratory investigation is known. - Complete the signature and initial box on the opposite page - Look at the care planned for your patient as set out in the ICP. - Decide if this care is appropriate for your patient. - If yes, then deliver the care, record the time it was completed, and initial the relevant box. - If you decide to change the care from what is set out in the ICP, then you must record a *‘variance’.* - *To record a variance you must write next to the activity concerned* - the time the variance occurred - what the variance was using the codes on the back of the pathway - what action you took instead of that planned - initial the variance recording - If more space is required to record the variance or additional notes, continue on the multidisciplinary note sheet using the appropriate code as a reference point. - **Always** use black ink in filling out this record. - All Nursing Assessment and Care should be recorded in this ICP and not on the Nursing Information System (NIS). If any other problems are identified during and after the assessment process, which are not reflected within this pathway, then a care plan should be created on the NIS   **Where should the ICP be kept?**  Patient’s Medical Records folder  Who can I contact for more information? For more information about Treatment of Cutaneous Leishmaniasis or ICP’s please contact  - T8 Ward Sister/Charge Nurse - Modern Matron, Directorate of Infection - Practice Development Facilitator T8 | | | |

| Day Case – Cut Leishmaniasis ICP | | | | AFFIX PATIENT ID LABEL HERE | | | | | Date |  | |
| --- | --- | --- | --- | --- | --- | --- | --- | --- | --- | --- | --- |
| MEDICAL | **Code** | ACTION | | | Time | **Sign** | | **Reason for variance & action taken (use codes)** | | | **Sign** |
|  | **1^st^ consultation - Investigations** | | | | | | | | | | |
|  | **M1** | Patient consented to investigations | | |  |  | |  | | |  |
|  | **M2** | Biopsy taken and sent to Histopathology | | |  |  | |  | | |  |
|  | **M3** | Biopsy taken and sent to Parasitology, and Culture and PCR arranged | | |  |  | |  | | |  |
|  | **M4** | Slit skin smear taken and sent | | |  |  | |  | | |  |
|  | **M5** | FBC taken and sent | | |  |  | |  | | |  |
|  | **M6** | LFT taken and sent | | |  |  | |  | | |  |
|  | **M7** | U & E taken and sent | | |  |  | |  | | |  |
|  | **M8** | Treatment options discussed with the patient | | |  |  | |  | | |  |
|  | **M9** | Information leaflet given (including out of hours contact details) | | |  |  | |  | | |  |
|  | **Code** | ACTION | | | Time | **Sign** | | **Reason for variance & action taken (use codes)** | | | **Sign** |
|  | **2^nd^ consultation** | | | | Date | | |  | | | |
|  | **M10** | Patient informed of diagnosis | | |  |  | |  | | |  |
|  | **M11** | Patient fits criteria for day treatment   - Under 65 years old □ - Able to attend daily for   21 / 28 days □   - No pre-existing cardiac or renal   condition □   - Normal FBC □ - Normal U & E □ - Normal LFt □ - Normal ECG □   Upper limit of QT interval: 0.420 seconds (420 milliseconds)  (see variance code on p. 55 if does not fit criteria) | | |  |  | |  | | |  |
|  | **M12** | Patient consented and admitted for daycase treatment | | |  |  | |  | | |  |
|  | **M13** | Informed Nurse on Floor 8 | | |  |  | |  | | |  |
| **Investigations** | | | | | | | | | | | |
| **Date** | | | **Investigation** | | | | **Result** | | | | |
|  | | |  | | | |  | | | | |
|  | | |  | | | |  | | | | |
|  | | |  | | | |  | | | | |
|  | | |  | | | |  | | | | |
|  | | |  | | | |  | | | | |
| **Screening Assessment Completed By:**  **Name (Print): Signature**  **Designation** | | | | | | | | | | | |

| Day Case - Cut Leishmaniasis ICP | | | AFFIX PATIENT ID LABEL HERE | Date | |  |
| --- | --- | --- | --- | --- | --- | --- |
| **DATE** | **TIME** | Multi-Disciplinary Notes | | | **SIGNATURE** | |
|  |  |  | | |  | |
|  |  |  | | |  | |
|  |  |  | | |  | |
|  |  |  | | |  | |
|  |  |  | | |  | |
|  |  |  | | |  | |
|  |  |  | | |  | |
|  |  |  | | |  | |
|  |  |  | | |  | |
|  |  |  | | |  | |
|  |  |  | | |  | |
|  |  |  | | |  | |
|  |  |  | | |  | |
|  |  |  | | |  | |
|  |  |  | | |  | |
|  |  |  | | |  | |
|  |  |  | | |  | |
|  |  |  | | |  | |
|  |  |  | | |  | |
|  |  |  | | |  | |
|  |  |  | | |  | |
|  |  |  | | |  | |
|  |  |  | | |  | |
|  |  |  | | |  | |
|  |  |  | | |  | |
|  |  |  | | |  | |
|  |  |  | | |  | |
|  |  |  | | |  | |
|  |  |  | | |  | |
|  |  |  | | |  | |
|  |  |  | | |  | |
|  |  |  | | |  | |
|  |  |  | | |  | |
|  |  |  | | |  | |
|  |  |  | | |  | |
|  |  |  | | |  | |
|  |  |  | | |  | |
|  |  |  | | |  | |
|  |  |  | | |  | |
|  |  |  | | |  | |
|  |  |  | | |  | |
|  |  |  | | |  | |
|  |  |  | | |  | |
|  |  |  | | |  | |
|  |  |  | | |  | |
|  |  |  | | |  | |
|  |  |  | | |  | |

| Day Case - Cut Leishmaniasis ICP | | | | AFFIX PATIENT ID LABEL HERE | | | | Date | |  | |
| --- | --- | --- | --- | --- | --- | --- | --- | --- | --- | --- | --- |
| NURSING | **Code** | ACTION | | | Time | **Sign** | **Reason for variance & action taken (use codes)** | | | | **Sign** |
|  | **Day 1** | | | | | | | | | | |
|  | **N1** | Confirmed patient fits criteria for day case treatment (ref M11) | | |  |  |  | | | |  |
|  | **N2** | Explain to the patient the clinic procedures (include drug information and possible side effects) | | |  |  |  | | | |  |
|  | N3 | Lesion assessment chart completed | | |  |  |  | | | |  |
|  | **N4** | Lesion dressed according to assessment and plan | | |  |  |  | | | |  |
|  | N5 | FBC taken and sent | | |  |  |  | | | |  |
|  | N6 | LFT taken and sent | | |  |  |  | | | |  |
|  | N7 | U & E taken and sent | | |  |  |  | | | |  |
|  | **N8** | ECG recorded | | |  |  |  | | | |  |
|  | **N9** | IV sodium stibogluconate administered (ref. p. 105) | | |  |  |  | | | |  |
|  | **N10** | Patient observed for any adverse events. (record below) | | |  |  |  | | | |  |
|  | **N11** | **Adverse Events** | | | | Action | | | | | |
|  |  | Nausea: yes □ no □ | | | |  | | | | | |
|  |  | Malaise: yes □ no □ | | | |  | | | | | |
|  |  | Abdominal pain: yes □ no □ | | | |  | | | | | |
|  |  | Myalgia: yes □ no □ | | | |  | | | | | |
|  |  | Skin rash: yes □ no □  Location: | | | |  | | | | | |
|  | **N12** | Patient reviewed by SHO or SpR | | |  |  |  | | | |  |
|  | **N13** | SHO/SpR informed of adverse events | | |  |  |  | | | |  |
|  | N14 | **Observations (before treatment)** | | |  |  |  | | | |  |
|  |  | Blood Pressure |  | | | Temperature | | |  | | |
|  |  | Respiration |  | | | Pulse | | |  | | |
|  |  | **Observations (during treatment)** | | |  |  |  | | | |  |
|  |  | Blood Pressure |  | | | Temperature | | |  | | |
|  |  | Respiration |  | | | Pulse | | |  | | |
|  |  | **Observations (after treatment)** | | |  |  |  | | | |  |
|  |  | Blood Pressure |  | | | Temperature | | |  | | |
|  |  | Respiration |  | | | Pulse | | |  | | |
|  |  | **Lesion** Ulcer □ Induration □ Scar/Epithelialising □ | | | | | | | | | |

| Day Case - Cut Leishmaniasis ICP | | | AFFIX PATIENT ID LABEL HERE | | Date | |  |  |
| --- | --- | --- | --- | --- | --- | --- | --- | --- |
| **DATE** | **TIME** | Multi-Disciplinary Notes | | | | **SIGNATURE** | |  |
|  |  |  | | | |  | |  |
|  |  |  | | | |  | |  |
|  |  |  | | | |  | |  |
|  |  |  | | | |  | |  |
|  |  |  | | | |  | |  |
|  |  |  | | | |  | |  |
|  |  |  | | | |  | |  |
|  |  |  | | | |  | |  |
|  |  |  | | | |  | |  |
|  |  |  | | | |  | |  |
|  |  |  | | | |  | |  |
|  |  |  | | | |  | |  |
|  |  |  | | | |  | |  |
|  |  |  | | | |  | |  |
|  |  |  | | | |  | |  |
|  |  |  | | | |  | |  |
|  |  |  | | | |  | |  |
|  |  |  | | | |  | |  |
|  |  |  | | | |  | |  |
|  |  |  | | | |  | |  |
|  |  |  | | | |  | |  |
|  |  |  | | | |  | |  |
|  |  |  | | | |  | |  |
|  |  |  | | | |  | |  |
|  |  |  | | | |  | |  |
|  |  |  | | | |  | |  |
|  |  |  | | | |  | |  |
|  |  |  | | | |  | |  |
|  |  |  | | | |  | |  |
|  |  |  | | | |  | |  |
|  |  |  | | | |  | |  |
|  |  |  | | | |  | |  |
|  |  |  | | | |  | |  |
|  |  |  | | | |  | |  |
|  |  |  | | | |  | |  |
|  |  |  | | | |  | |  |
|  |  |  | | | |  | |  |
|  |  |  | | | |  | |  |
|  |  |  | | | |  | |  |
|  |  |  | | | |  | |  |
|  |  |  | | | |  | |  |
|  |  |  | | | |  | |  |
|  |  |  | | | |  | |  |
|  |  |  | | | |  | |  |
|  |  |  | | | |  | |  |
|  |  |  | | | |  | |  |
|  |  |  | | | |  | |  |
| 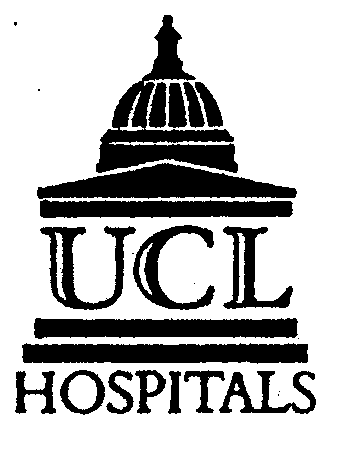  ***Assessment of Lesion***  ***Cutaneous Leishmaniasis***  Date: __________________________________ | | | | Attach Patient Identification Sticker or complete  **PATIENT NAME**  __________________________________________  HOSPITAL NUMBER __________________________________________ | | | | |

***ssment*** (Nurse to complete)

| **LESION GRADE (From lesion and pressure area care policy) 0 1 2 3 4** | **INFECTION** Swab sent **YES / NO** Date ________ |
| --- | --- |
| **LESION FLOOR CONDITION**  Healthy Granulation **YES / NO**  Thick Slough (Yellow / Brown) **YES / NO** | **RESULT**: Date:________ |
| Necrotic (Black) **YES /NO**  Cellulitis **YES / NO** | **ODOUR YES / NO** |
| **EXUDATE**  Colour  Amount | **PAIN** None  Dressing changes only |
| **CONDITION OF SURROUNDING SKIN**  e.g. Blisters, Fragile, etc. | Continuous  Name of analgesic: |

***Planning*  (Nurse to complete)**

| Debridement Method: |
| --- |
| Cleansing Solution: |
| Topical Agent / Dressing required: |
| Amount of dressing required per dressing change / size of dressing needed (Approx) |
| Frequency of dressing change per day and rationale (e.g. as per protocol) |
| LESION ASSESSED BY (Print & Sign) ________________________ GRADE ___________Review Date __________ |

**PHARMACY DRESSING SUPPLY (Pharmacist to complete)**

| **Dressing** |  |  |  |  |  |
| --- | --- | --- | --- | --- | --- |
| **Quantity supplied** |  |  |  |  |  |
| **Date supplied** |  |  |  |  |  |
| **Signature of Pharmacist** |  |  |  |  |  |

| Day Case - Cut Leishmaniasis ICP | | | | | | AFFIX PATIENT ID LABEL HERE | | | | Date | | |  | |
| --- | --- | --- | --- | --- | --- | --- | --- | --- | --- | --- | --- | --- | --- | --- |
| NURSING | **Code** | | ACTION | | | | Time | **Sign** | **Reason for variance & action taken (use codes)** | | | | | **Sign** |
|  | **Day 2** | | | | | | | | | | | | | |
|  | **N15** | | Lesion redressed according to plan | | | |  |  |  | | | | |  |
|  | **N16** | | IV sodium stibogluconate administered (ref. p. 105) | | | |  |  |  | | | | |  |
|  | **N17** | | Patient observed for any adverse events (record below) | | | |  |  |  | | | | |  |
|  | **N18** | | **Adverse Events** | | | | | **Action taken** | | | | | | |
|  |  |  | Nausea: yes □ no □ | | | | |  | | | | | | |
|  |  |  | Malaise: yes □ no □ | | | | |  | | | | | | |
|  |  |  | Abdominal pain: yes □ no □ | | | | |  | | | | | | |
|  |  |  | Myalgia: yes □ no □ | | | | |  | | | | | | |
|  |  |  | Skin rash: yes □ no □  Location: | | | | |  | | | | | | |
|  | **N19** | | Observations (before treatment) | | | |  |  |  | | | | |  |
|  |  |  | Blood pressure | |  | | | Temperature | | |  | | | |
|  |  |  | Respiration | |  | | | Pulse | | |  | | | |
|  |  |  | Observations (during treatment) | | | |  |  |  | | | | |  |
|  |  |  | Blood pressure | |  | | | Temperature | | |  | | | |
|  |  |  | Respiration | |  | | | Pulse | | |  | | | |
|  |  |  | Observations (after treatment) | | | |  |  |  | | | | |  |
|  |  |  | Blood pressure | |  | | | Temperature | | |  | | | |
|  |  |  | Respiration | |  | | | Pulse | | |  | | | |
| **DATE** | | **TIME** | | Multi-Disciplinary Notes | | | | | | | | **SIGNATURE** | | |
|  | |  | |  | | | | | | | |  | | |
|  | |  | |  | | | | | | | |  | | |
|  | |  | |  | | | | | | | |  | | |
|  | |  | |  | | | | | | | |  | | |
|  | |  | |  | | | | | | | |  | | |
|  | |  | |  | | | | | | | |  | | |
|  | |  | |  | | | | | | | |  | | |
|  | |  | |  | | | | | | | |  | | |
|  | |  | |  | | | | | | | |  | | |
|  | |  | |  | | | | | | | |  | | |
|  | |  | |  | | | | | | | |  | | |
|  | |  | |  | | | | | | | |  | | |
|  | |  | |  | | | | | | | |  | | |
|  | |  | |  | | | | | | | |  | | |
|  | |  | |  | | | | | | | |  | | |
|  | |  | |  | | | | | | | |  | | |

| Day Case - Cut Leishmaniasis ICP | | | | | | AFFIX PATIENT ID LABEL HERE | | | | Date | | |  | |
| --- | --- | --- | --- | --- | --- | --- | --- | --- | --- | --- | --- | --- | --- | --- |
| NURSING | **Code** | | ACTION | | | | Time | **Sign** | **Reason for variance & action taken (use codes)** | | | | | **Sign** |
|  | **Day 3** | | | | | | | | | | | | | |
|  | N20 | | Lesion redressed according to plan | | | |  |  |  | | | | |  |
|  | **N21** | | IV sodium stibogluconate administered (ref. p. 105) | | | |  |  |  | | | | |  |
|  | **N22** | | Patient observed for any adverse events (record below) | | | |  |  |  | | | | |  |
|  | **N23** | | **Adverse Events** | | | | | **Action taken** | | | | | | |
|  |  |  | Nausea: yes □ no □ | | | | |  | | | | | | |
|  |  |  | Malaise: yes □ no □ | | | | |  | | | | | | |
|  |  |  | Abdominal pain: yes □ no □ | | | | |  | | | | | | |
|  |  |  | Myalgia: yes □ no □ | | | | |  | | | | | | |
|  |  |  | Skin rash: yes □ no □  Location: | | | | |  | | | | | | |
|  | **N24** | | SHO/SpR informed of adverse events | | | |  |  |  | | | | |  |
|  | **N25** | | Observations (before treatment) | | | |  |  |  | | | | |  |
|  |  |  | Blood pressure | |  | | | Temperature | | |  | | | |
|  |  |  | Respiration | |  | | | Pulse | | |  | | | |
|  |  |  | Observations (during treatment) | | | |  |  |  | | | | |  |
|  |  |  | Blood pressure | |  | | | Temperature | | |  | | | |
|  |  |  | Respiration | |  | | | Pulse | | |  | | | |
|  |  |  | Observations (after treatment) | | | |  |  |  | | | | |  |
|  |  |  | Blood pressure | |  | | | Temperature | | |  | | | |
|  |  |  | Respiration | |  | | | Pulse | | |  | | | |
| **DATE** | | **TIME** | | Multi-Disciplinary Notes | | | | | | | | **SIGNATURE** | | |
|  | |  | |  | | | | | | | |  | | |
|  | |  | |  | | | | | | | |  | | |
|  | |  | |  | | | | | | | |  | | |
|  | |  | |  | | | | | | | |  | | |
|  | |  | |  | | | | | | | |  | | |
|  | |  | |  | | | | | | | |  | | |
|  | |  | |  | | | | | | | |  | | |
|  | |  | |  | | | | | | | |  | | |
|  | |  | |  | | | | | | | |  | | |
|  | |  | |  | | | | | | | |  | | |
|  | |  | |  | | | | | | | |  | | |
|  | |  | |  | | | | | | | |  | | |
|  | |  | |  | | | | | | | |  | | |
|  | |  | |  | | | | | | | |  | | |
|  | |  | |  | | | | | | | |  | | |
|  | |  | |  | | | | | | | |  | | |
|  | |  | |  | | | | | | | |  | | |
|  | |  | |  | | | | | | | |  | | |

| Day Case - Cut Leishmaniasis ICP | | | | | | AFFIX PATIENT ID LABEL HERE | | | | Date | | |  | |
| --- | --- | --- | --- | --- | --- | --- | --- | --- | --- | --- | --- | --- | --- | --- |
| NURSING | **Code** | | ACTION | | | | Time | **Sign** | **Reason for variance & action taken (use codes)** | | | | | **Sign** |
|  | **Day 4** | | | | | | | | | | | | | |
|  | **N26** | | Lesion redressed according to plan | | | |  |  |  | | | | |  |
|  | N27 | | FBC taken and sent | | | |  |  |  | | | | |  |
|  | N28 | | LFT taken and sent | | | |  |  |  | | | | |  |
|  | N29 | | U & E taken and sent | | | |  |  |  | | | | |  |
|  | N30 | | ECG recorded | | | |  |  |  | | | | |  |
|  | **N31** | | IV sodium stibogluconate administered (ref. p. 105) | | | |  |  |  | | | | |  |
|  | **N32** | | Patient observed for any adverse events (record below) | | | |  |  |  | | | | |  |
|  | **N33** | | **Adverse Events** | | | | | **Action taken** | | | | | | |
|  |  |  | Nausea: yes □ no □ | | | | |  | | | | | | |
|  |  |  | Malaise: yes □ no □ | | | | |  | | | | | | |
|  |  |  | Abdominal pain: yes □ no □ | | | | |  | | | | | | |
|  |  |  | Myalgia: yes □ no □ | | | | |  | | | | | | |
|  |  |  | Skin rash: yes □ no □  Location: | | | | |  | | | | | | |
|  | **N34** | | Patient reviewed by SHO or SpR | | | |  |  |  | | | | |  |
|  | **N35** | | **Observations (before treatment)** | | | |  |  |  | | | | |  |
|  |  |  | Blood pressure | |  | | | Temperature | | |  | | | |
|  |  |  | Respiration | |  | | | Pulse | | |  | | | |
|  |  |  | **Observations (during treatment)** | | | |  |  |  | | | | |  |
|  |  |  | Blood pressure | |  | | | Temperature | | |  | | | |
|  |  |  | Respiration | |  | | | Pulse | | |  | | | |
|  |  |  | **Observations (after treatment)** | | | |  |  |  | | | | |  |
|  |  |  | Blood pressure | |  | | | Temperature | | |  | | | |
|  |  |  | Respiration | |  | | | Pulse | | |  | | | |
|  |  |  | **Lesion**  Ulcer □ Induration □ Scar/Epithelialising □ | | | | | | | | | | | |
| **DATE** | | **TIME** | | Multi-Disciplinary Notes | | | | | | | | **SIGNATURE** | | |
|  | |  | |  | | | | | | | |  | | |
|  | |  | |  | | | | | | | |  | | |
|  | |  | |  | | | | | | | |  | | |
|  | |  | |  | | | | | | | |  | | |
|  | |  | |  | | | | | | | |  | | |
|  | |  | |  | | | | | | | |  | | |
|  | |  | |  | | | | | | | |  | | |
|  | |  | |  | | | | | | | |  | | |
|  | |  | |  | | | | | | | |  | | |
|  | |  | |  | | | | | | | |  | | |
|  | |  | |  | | | | | | | |  | | |
|  | |  | |  | | | | | | | |  | | |

| Day Case - Cut Leishmaniasis ICP | | | | | | AFFIX PATIENT ID LABEL HERE | | | | Date | | |  | |
| --- | --- | --- | --- | --- | --- | --- | --- | --- | --- | --- | --- | --- | --- | --- |
| NURSING | **Code** | | ACTION | | | | Time | **Sign** | **Reason for variance & action taken (use codes)** | | | | | **Sign** |
|  | **Day 5** | | | | | | | | | | | | | |
|  | N36 | | Lesion redressed according to plan | | | |  |  |  | | | | |  |
|  | **N37** | | IV sodium stibogluconate administered (ref. p. 105) | | | |  |  |  | | | | |  |
|  | **N38** | | Patient observed for any adverse events (record below) | | | |  |  |  | | | | |  |
|  | **N39** | | **Adverse Events** | | | | | **Action taken** | | | | | | |
|  |  |  | Nausea: yes □ no □ | | | | |  | | | | | | |
|  |  |  | Malaise: yes □ no □ | | | | |  | | | | | | |
|  |  |  | Abdominal pain: yes □ no □ | | | | |  | | | | | | |
|  |  |  | Myalgia: yes □ no □ | | | | |  | | | | | | |
|  |  |  | Skin rash: yes □ no □  Location: | | | | |  | | | | | | |
|  | **N40** | | SHO/SpR informed of adverse events | | | |  |  |  | | | | |  |
|  | **N41** | | Observations (before treatment) | | | |  |  |  | | | | |  |
|  |  |  | Blood pressure | |  | | | Temperature | | |  | | | |
|  |  |  | Respiration | |  | | | Pulse | | |  | | | |
|  |  |  | Observations (during treatment) | | | |  |  |  | | | | |  |
|  |  |  | Blood pressure | |  | | | Temperature | | |  | | | |
|  |  |  | Respiration | |  | | | Pulse | | |  | | | |
|  |  |  | Observations (after treatment) | | | |  |  |  | | | | |  |
|  |  |  | Blood pressure | |  | | | Temperature | | |  | | | |
|  |  |  | Respiration | |  | | | Pulse | | |  | | | |
| **DATE** | | **TIME** | | Multi-Disciplinary Notes | | | | | | | | **SIGNATURE** | | |
|  | |  | |  | | | | | | | |  | | |
|  | |  | |  | | | | | | | |  | | |
|  | |  | |  | | | | | | | |  | | |
|  | |  | |  | | | | | | | |  | | |
|  | |  | |  | | | | | | | |  | | |
|  | |  | |  | | | | | | | |  | | |
|  | |  | |  | | | | | | | |  | | |
|  | |  | |  | | | | | | | |  | | |
|  | |  | |  | | | | | | | |  | | |
|  | |  | |  | | | | | | | |  | | |
|  | |  | |  | | | | | | | |  | | |
|  | |  | |  | | | | | | | |  | | |
|  | |  | |  | | | | | | | |  | | |
|  | |  | |  | | | | | | | |  | | |
|  | |  | |  | | | | | | | |  | | |
|  | |  | |  | | | | | | | |  | | |
|  | |  | |  | | | | | | | |  | | |
|  | |  | |  | | | | | | | |  | | |
|  | |  | |  | | | | | | | |  | | |
| Day Case - Cut Leishmaniasis ICP | | | | | | AFFIX PATIENT ID LABEL HERE | | | | Date | | |  | |
| NURSING | **Code** | | ACTION | | | | Time | **Sign** | **Reason for variance & action taken (use codes)** | | | | | **Sign** |
|  | **Day 6** | | | | | | | | | | | | | |
|  | **N42** | | Lesion redressed according to plan | | | |  |  |  | | | | |  |
|  | **N43** | | IV sodium stibogluconate administered (ref. p. 105) | | | |  |  |  | | | | |  |
|  | **N44** | | Patient observed for any adverse events (record below) | | | |  |  |  | | | | |  |
|  | **N45** | | **Adverse Events** | | | | | **Action taken** | | | | | | |
|  |  |  | Nausea: yes □ no □ | | | | |  | | | | | | |
|  |  |  | Malaise: yes □ no □ | | | | |  | | | | | | |
|  |  |  | Abdominal pain: yes □ no □ | | | | |  | | | | | | |
|  |  |  | Myalgia: yes □ no □ | | | | |  | | | | | | |
|  |  |  | Skin rash: yes □ no □  Location: | | | | |  | | | | | | |
|  | **N46** | | SHO/SpR informed of adverse events | | | |  |  |  | | | | |  |
|  | **N47** | | Observations (before treatment) | | | |  |  |  | | | | |  |
|  |  |  | Blood pressure | |  | | | Temperature | | |  | | | |
|  |  |  | Respiration | |  | | | Pulse | | |  | | | |
|  |  |  | Observations (during treatment) | | | |  |  |  | | | | |  |
|  |  |  | Blood pressure | |  | | | Temperature | | |  | | | |
|  |  |  | Respiration | |  | | | Pulse | | |  | | | |
|  |  |  | Observations (after treatment) | | | |  |  |  | | | | |  |
|  |  |  | Blood pressure | |  | | | Temperature | | |  | | | |
|  |  |  | Respiration | |  | | | Pulse | | |  | | | |
| **DATE** | | **TIME** | | Multi-Disciplinary Notes | | | | | | | | **SIGNATURE** | | |
|  | |  | |  | | | | | | | |  | | |
|  | |  | |  | | | | | | | |  | | |
|  | |  | |  | | | | | | | |  | | |
|  | |  | |  | | | | | | | |  | | |
|  | |  | |  | | | | | | | |  | | |
|  | |  | |  | | | | | | | |  | | |
|  | |  | |  | | | | | | | |  | | |
|  | |  | |  | | | | | | | |  | | |
|  | |  | |  | | | | | | | |  | | |
|  | |  | |  | | | | | | | |  | | |
|  | |  | |  | | | | | | | |  | | |
|  | |  | |  | | | | | | | |  | | |
|  | |  | |  | | | | | | | |  | | |
|  | |  | |  | | | | | | | |  | | |
|  | |  | |  | | | | | | | |  | | |
|  | |  | |  | | | | | | | |  | | |
|  | |  | |  | | | | | | | |  | | |
|  | |  | |  | | | | | | | |  | | |

| Day Case - Cut Leishmaniasis ICP | | | | | | AFFIX PATIENT ID LABEL HERE | | | | Date | | |  | |
| --- | --- | --- | --- | --- | --- | --- | --- | --- | --- | --- | --- | --- | --- | --- |
| NURSING | **Code** | | ACTION | | | | Time | **Sign** | **Reason for variance & action taken (use codes)** | | | | | **Sign** |
|  | **Day 7** | | | | | | | | | | | | | |
|  | N48 | | Lesion reassessment and new chart completed (p21) | | | |  |  |  | | | | |  |
|  | **N49** | | Lesion redressed according to reassessment and plan | | | |  |  |  | | | | |  |
|  | N50 | | FBC taken and sent | | | |  |  |  | | | | |  |
|  | N51 | | LFT taken and sent | | | |  |  |  | | | | |  |
|  | N52 | | U & E taken and sent | | | |  |  |  | | | | |  |
|  | N53 | | ECG recorded | | | |  |  |  | | | | |  |
|  | **N54** | | IV sodium stibogluconate administered (ref. p. 105) | | | |  |  |  | | | | |  |
|  | **N55** | | Patient observed for any adverse events | | | |  |  |  | | | | |  |
|  | **N56** | | **Adverse Events** | | | | | **Action taken** | | | | | | |
|  |  |  | Nausea: yes □ no □ | | | | |  | | | | | | |
|  |  |  | Malaise: yes □ no □ | | | | |  | | | | | | |
|  |  |  | Abdominal pain: yes □ no □ | | | | |  | | | | | | |
|  |  |  | Myalgia: yes □ no □ | | | | |  | | | | | | |
|  |  |  | Skin rash: yes □ no □  Location: | | | | |  | | | | | | |
|  | **N57** | | Patient reviewed by SHO or SpR | | | |  |  |  | | | | |  |
|  | N58 | | **Observations (before treatment)** | | | |  |  |  | | | | |  |
|  |  |  | Blood pressure | |  | | | Temperature | | |  | | | |
|  |  |  | Respiration | |  | | | Pulse | | |  | | | |
|  |  |  | **Observations (during treatment)** | | | |  |  |  | | | | |  |
|  |  |  | Blood pressure | |  | | | Temperature | | |  | | | |
|  |  |  | Respiration | |  | | | Pulse | | |  | | | |
|  |  |  | **Observations (after treatment)** | | | |  |  |  | | | | |  |
|  |  |  | Blood pressure | |  | | | Temperature | | |  | | | |
|  |  |  | Respiration | |  | | | Pulse | | |  | | | |
|  |  |  | **Lesion** Ulcer □ Induration □ Scar/Epithelialising □ | | | | | | | | | | | |
| **DATE** | | **TIME** | | Multi-Disciplinary Notes | | | | | | | | **SIGNATURE** | | |
|  | |  | |  | | | | | | | |  | | |
|  | |  | |  | | | | | | | |  | | |
|  | |  | |  | | | | | | | |  | | |
|  | |  | |  | | | | | | | |  | | |
|  | |  | |  | | | | | | | |  | | |
|  | |  | |  | | | | | | | |  | | |
|  | |  | |  | | | | | | | |  | | |
|  | |  | |  | | | | | | | |  | | |
|  | |  | |  | | | | | | | |  | | |
|  | |  | |  | | | | | | | |  | | |
|  | |  | |  | | | | | | | |  | | |

| 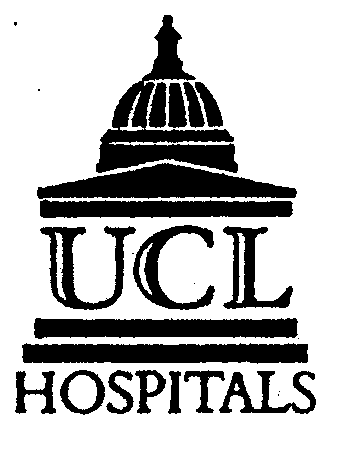  ***Assessment of Lesion***  ***Cutaneous Leishmaniasis***  Date: __________________________________ | | Attach Patient Identification Sticker or complete  **PATIENT NAME**  __________________________________________  HOSPITAL NUMBER __________________________________________ | |
| --- | --- | --- | --- |
| ***LOCATION OF LESION***  ***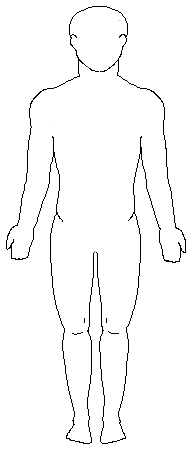*** | ***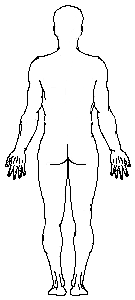*** | | ***DIAGRAM OF LESION***  (DIMENSIONS IN CM) |

***Initial assessment*** (Nurse to complete)

| **LESION GRADE (From lesion and pressure area care policy) 0 1 2 3 4** | **INFECTION** Swab sent **YES / NO** Date ________ |
| --- | --- |
| **LESION FLOOR CONDITION**  Healthy Granulation **YES / NO**  Thick Slough (Yellow / Brown) **YES / NO** | **RESULT**: Date:________ |
| Necrotic (Black) **YES /NO**  Cellulitis **YES / NO** | **ODOUR YES / NO** |
| **EXUDATE**  Colour  Amount | **PAIN** None  Dressing changes only |
| **CONDITION OF SURROUNDING SKIN**  e.g. Blisters, Fragile, etc. | Continuous  Name of analgesic: |

***Planning*  (Nurse to complete)**

| Debridement Method: |
| --- |
| Cleansing Solution: |
| Topical Agent / Dressing required: |
| Amount of dressing required per dressing change / size of dressing needed (Approx) |
| Frequency of dressing change per day and rationale (e.g. as per protocol) |
| LESION ASSESSED BY (Print & Sign) ________________________ GRADE ___________Review Date __________ |

**PHARMACY DRESSING SUPPLY** (Pharmacist to complete)

| **Dressing** | | | | | |  | | |  | | |  | | |  | | | |  | |
| --- | --- | --- | --- | --- | --- | --- | --- | --- | --- | --- | --- | --- | --- | --- | --- | --- | --- | --- | --- | --- |
| **Quantity supplied** | | | | | |  | | |  | | |  | | |  | | | |  | |
| **Date supplied** | | | | | |  | | |  | | |  | | |  | | | |  | |
| **Signature of Pharmacist** | | | | | |  | | |  | | |  | | |  | | | |  | |
| Day Case - Cut Leishmaniasis ICP | | | | | | | AFFIX PATIENT ID LABEL HERE | | | | | | Date | | | |  | | |  |
| NURSING | **Code** | | ACTION | | | | | Time | | **Sign** | **Reason for variance & action taken (use codes)** | | | | | | | **Sign** | |  |
|  | **Day 8** | | | | | | | | | | | | | | | | | | |  |
|  | N59 | | Lesion redressed according to plan | | | | |  | |  |  | | | | | | |  | |  |
|  | **N60** | | IV sodium stibogluconate administered (ref. p. 105) | | | | |  | |  |  | | | | | | |  | |  |
|  | **N61** | | Patient observed for any adverse events (record below) | | | | |  | |  |  | | | | | | |  | |  |
|  | **N62** | | **Adverse Events** | | | | | | | **Action taken** | | | | | | | | | |  |
|  |  |  | Nausea: yes □ no □ | | | | | | |  | | | | | | | | | |  |
|  |  |  | Malaise: yes □ no □ | | | | | | |  | | | | | | | | | |  |
|  |  |  | Abdominal pain: yes □ no □ | | | | | | |  | | | | | | | | | |  |
|  |  |  | Myalgia: yes □ no □ | | | | | | |  | | | | | | | | | |  |
|  |  |  | Skin rash: yes □ no □  Location: | | | | | | |  | | | | | | | | | |  |
|  | **N63** | | SHO/SpR informed of adverse events | | | | |  | |  |  | | | | | | |  | |  |
|  | **N64** | | Observations (before treatment) | | | | |  | |  |  | | | | | | |  | |  |
|  |  |  | Blood pressure | |  | | | | | Temperature | | | |  | | | | | |  |
|  |  |  | Respiration | |  | | | | | Pulse | | | |  | | | | | |  |
|  |  |  | Observations (during treatment) | | | | |  | |  |  | | | | | | |  | |  |
|  |  |  | Blood pressure | |  | | | | | Temperature | | | |  | | | | | |  |
|  |  |  | Respiration | |  | | | | | Pulse | | | |  | | | | | |  |
|  |  |  | Observations (after treatment) | | | | |  | |  |  | | | | | | |  | |  |
|  |  |  | Blood pressure | |  | | | | | Temperature | | | |  | | | | | |  |
|  |  |  | Respiration | |  | | | | | Pulse | | | |  | | | | | |  |
| DATE | | **TIME** | | Multi-Disciplinary Notes | | | | | | | | | | | | **SIGNATURE** | | | |  |
|  | |  | |  | | | | | | | | | | | |  | | | |  |
|  | |  | |  | | | | | | | | | | | |  | | | |  |
|  | |  | |  | | | | | | | | | | | |  | | | |  |
|  | |  | |  | | | | | | | | | | | |  | | | |  |
|  | |  | |  | | | | | | | | | | | |  | | | |  |
|  | |  | |  | | | | | | | | | | | |  | | | |  |
|  | |  | |  | | | | | | | | | | | |  | | | |  |
|  | |  | |  | | | | | | | | | | | |  | | | |  |
|  | |  | |  | | | | | | | | | | | |  | | | |  |
|  | |  | |  | | | | | | | | | | | |  | | | |  |
|  | |  | |  | | | | | | | | | | | |  | | | |  |
|  | |  | |  | | | | | | | | | | | |  | | | |  |
|  | |  | |  | | | | | | | | | | | |  | | | |  |
|  | |  | |  | | | | | | | | | | | |  | | | |  |
|  | |  | |  | | | | | | | | | | | |  | | | |  |
|  | |  | |  | | | | | | | | | | | |  | | | |  |
|  | |  | |  | | | | | | | | | | | |  | | | |  |
|  | |  | |  | | | | | | | | | | | |  | | | |  |
|  | |  | |  | | | | | | | | | | | |  | | | |  |

| Day Case - Cut Leishmaniasis ICP | | | | | | AFFIX PATIENT ID LABEL HERE | | | | Date | | |  | |
| --- | --- | --- | --- | --- | --- | --- | --- | --- | --- | --- | --- | --- | --- | --- |
| NURSING | **Code** | | ACTION | | | | Time | **Sign** | **Reason for variance & action taken (use codes)** | | | | | **Sign** |
|  | **Day 9** | | | | | | | | | | | | | |
|  | N65 | | Lesion redressed according to plan | | | |  |  |  | | | | |  |
|  | **N66** | | IV sodium stibogluconate administered (ref. p. 105) | | | |  |  |  | | | | |  |
|  | **N67** | | Patient observed for any adverse events (record below) | | | |  |  |  | | | | |  |
|  | **N68** | | **Adverse Events** | | | | | **Action taken** | | | | | | |
|  |  |  | Nausea: yes □ no □ | | | | |  | | | | | | |
|  |  |  | Malaise: yes □ no □ | | | | |  | | | | | | |
|  |  |  | Abdominal pain: yes □ no □ | | | | |  | | | | | | |
|  |  |  | Myalgia: yes □ no □ | | | | |  | | | | | | |
|  |  |  | Skin rash: yes □ no □  Location: | | | | |  | | | | | | |
|  | **N69** | | SHO/SpR informed of adverse events | | | |  |  |  | | | | |  |
|  | **N70** | | Observations (before treatment) | | | |  |  |  | | | | |  |
|  |  |  | Blood pressure | |  | | | Temperature | | |  | | | |
|  |  |  | Respiration | |  | | | Pulse | | |  | | | |
|  |  |  | Observations (during treatment) | | | |  |  |  | | | | |  |
|  |  |  | Blood pressure | |  | | | Temperature | | |  | | | |
|  |  |  | Respiration | |  | | | Pulse | | |  | | | |
|  |  |  | Observations (after treatment) | | | |  |  |  | | | | |  |
|  |  |  | Blood pressure | |  | | | Temperature | | |  | | | |
|  |  |  | Respiration | |  | | | Pulse | | |  | | | |
| DATE | | **TIME** | | Multi-Disciplinary Notes | | | | | | | | **SIGNATURE** | | |
|  | |  | |  | | | | | | | |  | | |
|  | |  | |  | | | | | | | |  | | |
|  | |  | |  | | | | | | | |  | | |
|  | |  | |  | | | | | | | |  | | |
|  | |  | |  | | | | | | | |  | | |
|  | |  | |  | | | | | | | |  | | |
|  | |  | |  | | | | | | | |  | | |
|  | |  | |  | | | | | | | |  | | |
|  | |  | |  | | | | | | | |  | | |
|  | |  | |  | | | | | | | |  | | |
|  | |  | |  | | | | | | | |  | | |
|  | |  | |  | | | | | | | |  | | |
|  | |  | |  | | | | | | | |  | | |
|  | |  | |  | | | | | | | |  | | |
|  | |  | |  | | | | | | | |  | | |
|  | |  | |  | | | | | | | |  | | |
|  | |  | |  | | | | | | | |  | | |
|  | |  | |  | | | | | | | |  | | |

| Day Case - Cut Leishmaniasis ICP | | | | | | AFFIX PATIENT ID LABEL HERE | | | | Date | | |  | |
| --- | --- | --- | --- | --- | --- | --- | --- | --- | --- | --- | --- | --- | --- | --- |
| NURSING | **Code** | | ACTION | | | | Time | **Sign** | **Reason for variance & action taken (use codes)** | | | | | **Sign** |
|  | **Day 10** | | | | | | | | | | | | | |
|  | **N71** | | Lesion redressed according to plan | | | |  |  |  | | | | |  |
|  | N72 | | FBC taken and sent | | | |  |  |  | | | | |  |
|  | N73 | | LFT taken and sent | | | |  |  |  | | | | |  |
|  | N74 | | U & E taken and sent | | | |  |  |  | | | | |  |
|  | N75 | | ECG recorded | | | |  |  |  | | | | |  |
|  | **N76** | | IV sodium stibogluconate administered (ref. p. 105) | | | |  |  |  | | | | |  |
|  | **N77** | | Patient observed for any adverse events (record below) | | | |  |  |  | | | | |  |
|  | **N78** | | **Adverse Events** | | | | | **Action taken** | | | | | | |
|  |  |  | Nausea: yes □ no □ | | | | |  | | | | | | |
|  |  |  | Malaise: yes □ no □ | | | | |  | | | | | | |
|  |  |  | Abdominal pain: yes □ no □ | | | | |  | | | | | | |
|  |  |  | Myalgia: yes □ no □ | | | | |  | | | | | | |
|  |  |  | Skin rash: yes □ no □  Location: | | | | |  | | | | | | |
|  | **N79** | | Patient reviewed by SHO or SpR | | | |  |  |  | | | | |  |
|  | **N80** | | **Observations (before treatment)** | | | |  |  |  | | | | |  |
|  |  |  | Blood pressure | |  | | | Temperature | | |  | | | |
|  |  |  | Respiration | |  | | | Pulse | | |  | | | |
|  |  |  | **Observations (during treatment)** | | | |  |  |  | | | | |  |
|  |  |  | Blood pressure | |  | | | Temperature | | |  | | | |
|  |  |  | Respiration | |  | | | Pulse | | |  | | | |
|  |  |  | **Observations (after treatment)** | | | |  |  |  | | | | |  |
|  |  |  | Blood pressure | |  | | | Temperature | | |  | | | |
|  |  |  | Respiration | |  | | | Pulse | | |  | | | |
|  |  |  | **Lesion** Ulcer □ Induration □ Scar/Epithelialising □ | | | | | | | | | | | |
| **DATE** | | **TIME** | | Multi-Disciplinary Notes | | | | | | | | **SIGNATURE** | | |
|  | |  | |  | | | | | | | |  | | |
|  | |  | |  | | | | | | | |  | | |
|  | |  | |  | | | | | | | |  | | |
|  | |  | |  | | | | | | | |  | | |
|  | |  | |  | | | | | | | |  | | |
|  | |  | |  | | | | | | | |  | | |
|  | |  | |  | | | | | | | |  | | |
|  | |  | |  | | | | | | | |  | | |
|  | |  | |  | | | | | | | |  | | |
|  | |  | |  | | | | | | | |  | | |
|  | |  | |  | | | | | | | |  | | |
|  | |  | |  | | | | | | | |  | | |
| Day Case - Cut Leishmaniasis ICP | | | | | | AFFIX PATIENT ID LABEL HERE | | | | Date | | |  | |
| NURSING | **Code** | | ACTION | | | | Time | **Sign** | **Reason for variance & action taken (use codes)** | | | | | **Sign** |
|  | **Day 11** | | | | | | | | | | | | | |
|  | N81 | | Lesion redressed according to plan | | | |  |  |  | | | | |  |
|  | **N82** | | IV sodium stibogluconate administered (ref. p. 105) | | | |  |  |  | | | | |  |
|  | **N83** | | Patient observed for any adverse events (record below) | | | |  |  |  | | | | |  |
|  | **N84** | | **Adverse Events** | | | | | **Action taken** | | | | | | |
|  |  |  | Nausea: yes □ no □ | | | | |  | | | | | | |
|  |  |  | Malaise: yes □ no □ | | | | |  | | | | | | |
|  |  |  | Abdominal pain: yes □ no □ | | | | |  | | | | | | |
|  |  |  | Myalgia: yes □ no □ | | | | |  | | | | | | |
|  |  |  | Skin rash: yes □ no □  Location: | | | | |  | | | | | | |
|  | **N85** | | SHO/SpR informed of adverse events | | | |  |  |  | | | | |  |
|  | **N86** | | Observations (before treatment) | | | |  |  |  | | | | |  |
|  |  |  | Blood pressure | |  | | | Temperature | | |  | | | |
|  |  |  | Respiration | |  | | | Pulse | | |  | | | |
|  |  |  | Observations (during treatment) | | | |  |  |  | | | | |  |
|  |  |  | Blood pressure | |  | | | Temperature | | |  | | | |
|  |  |  | Respiration | |  | | | Pulse | | |  | | | |
|  |  |  | Observations (after treatment) | | | |  |  |  | | | | |  |
|  |  |  | Blood pressure | |  | | | Temperature | | |  | | | |
|  |  |  | Respiration | |  | | | Pulse | | |  | | | |
| **DATE** | | **TIME** | | Multi-Disciplinary Notes | | | | | | | | **SIGNATURE** | | |
|  | |  | |  | | | | | | | |  | | |
|  | |  | |  | | | | | | | |  | | |
|  | |  | |  | | | | | | | |  | | |
|  | |  | |  | | | | | | | |  | | |
|  | |  | |  | | | | | | | |  | | |
|  | |  | |  | | | | | | | |  | | |
|  | |  | |  | | | | | | | |  | | |
|  | |  | |  | | | | | | | |  | | |
|  | |  | |  | | | | | | | |  | | |
|  | |  | |  | | | | | | | |  | | |
|  | |  | |  | | | | | | | |  | | |
|  | |  | |  | | | | | | | |  | | |
|  | |  | |  | | | | | | | |  | | |
|  | |  | |  | | | | | | | |  | | |
|  | |  | |  | | | | | | | |  | | |
|  | |  | |  | | | | | | | |  | | |
|  | |  | |  | | | | | | | |  | | |
|  | |  | |  | | | | | | | |  | | |
|  | |  | |  | | | | | | | |  | | |
| Day Case - Cut Leishmaniasis ICP | | | | | | AFFIX PATIENT ID LABEL HERE | | | | Date | | |  | |
| NURSING | **Code** | | ACTION | | | | Time | **Sign** | **Reason for variance & action taken (use codes)** | | | | | **Sign** |
|  | **Day 12** | | | | | | | | | | | | | |
|  | N87 | | Lesion redressed according to plan | | | |  |  |  | | | | |  |
|  | **N88** | | IV sodium stibogluconate administered (ref. p. 105) | | | |  |  |  | | | | |  |
|  | **N89** | | Patient observed for any adverse events (record below) | | | |  |  |  | | | | |  |
|  | **N90** | | **Adverse Events** | | | | | **Action taken** | | | | | | |
|  |  |  | Nausea: yes □ no □ | | | | |  | | | | | | |
|  |  |  | Malaise: yes □ no □ | | | | |  | | | | | | |
|  |  |  | Abdominal pain: yes □ no □ | | | | |  | | | | | | |
|  |  |  | Myalgia: yes □ no □ | | | | |  | | | | | | |
|  |  |  | Skin rash: yes □ no □  Location: | | | | |  | | | | | | |
|  | **N91** | | SHO/SpR informed of adverse events | | | |  |  |  | | | | |  |
|  | **N92** | | Observations (before treatment) | | | |  |  |  | | | | |  |
|  |  |  | Blood pressure | |  | | | Temperature | | |  | | | |
|  |  |  | Respiration | |  | | | Pulse | | |  | | | |
|  |  |  | Observations (during treatment) | | | |  |  |  | | | | |  |
|  |  |  | Blood pressure | |  | | | Temperature | | |  | | | |
|  |  |  | Respiration | |  | | | Pulse | | |  | | | |
|  |  |  | Observations (after treatment) | | | |  |  |  | | | | |  |
|  |  |  | Blood pressure | |  | | | Temperature | | |  | | | |
|  |  |  | Respiration | |  | | | Pulse | | |  | | | |
| DATE | | **TIME** | | Multi-Disciplinary Notes | | | | | | | | **SIGNATURE** | | |
|  | |  | |  | | | | | | | |  | | |
|  | |  | |  | | | | | | | |  | | |
|  | |  | |  | | | | | | | |  | | |
|  | |  | |  | | | | | | | |  | | |
|  | |  | |  | | | | | | | |  | | |
|  | |  | |  | | | | | | | |  | | |
|  | |  | |  | | | | | | | |  | | |
|  | |  | |  | | | | | | | |  | | |
|  | |  | |  | | | | | | | |  | | |
|  | |  | |  | | | | | | | |  | | |
|  | |  | |  | | | | | | | |  | | |
|  | |  | |  | | | | | | | |  | | |
|  | |  | |  | | | | | | | |  | | |
|  | |  | |  | | | | | | | |  | | |
|  | |  | |  | | | | | | | |  | | |
|  | |  | |  | | | | | | | |  | | |
|  | |  | |  | | | | | | | |  | | |
|  | |  | |  | | | | | | | |  | | |
|  | |  | |  | | | | | | | |  | | |
| Day Case - Cut Leishmaniasis ICP | | | | | | AFFIX PATIENT ID LABEL HERE | | | | Date | | |  | |
| NURSING | **Code** | | ACTION | | | | Time | **Sign** | **Reason for variance & action taken (use codes)** | | | | | **Sign** |
|  | **Day 13** | | | | | | | | | | | | | |
|  | N93 | | Lesion reassessment chart completed (p34) | | | |  |  |  | | | | |  |
|  | **N94** | | Lesion redressed according to reassessment (p34) | | | |  |  |  | | | | |  |
|  | N95 | | FBC taken and sent | | | |  |  |  | | | | |  |
|  | N96 | | LFT taken and sent | | | |  |  |  | | | | |  |
|  | N97 | | U & E taken and sent | | | |  |  |  | | | | |  |
|  | N98 | | ECG recorded | | | |  |  |  | | | | |  |
|  | **N99** | | IV sodium stibogluconate administered (ref. p. 105) | | | |  |  |  | | | | |  |
|  | **N100** | | Patient observed for any adverse events (record below) | | | |  |  |  | | | | |  |
|  | **N101** | | **Adverse Events** | | | | | **Action taken** | | | | | | |
|  |  |  | Nausea: yes □ no □ | | | | |  | | | | | | |
|  |  |  | Malaise: yes □ no □ | | | | |  | | | | | | |
|  |  |  | Abdominal pain: yes □ no □ | | | | |  | | | | | | |
|  |  |  | Myalgia: yes □ no □ | | | | |  | | | | | | |
|  |  |  | Skin rash: yes □ no □  Location: | | | | |  | | | | | | |
|  | **N102** | | Patient reviewed by SHO or SpR | | | |  |  |  | | | | |  |
|  | **N103** | | Observations (before treatment) | | | |  |  |  | | | | |  |
|  |  |  | Blood pressure | |  | | | Temperature | | |  | | | |
|  |  |  | Respiration | |  | | | Pulse | | |  | | | |
|  |  |  | Observations (during treatment) | | | |  |  |  | | | | |  |
|  |  |  | Blood pressure | |  | | | Temperature | | |  | | | |
|  |  |  | Respiration | |  | | | Pulse | | |  | | | |
|  |  |  | Observations (after treatment) | | | |  |  |  | | | | |  |
|  |  |  | Blood pressure | |  | | | Temperature | | |  | | | |
|  |  |  | Respiration | |  | | | Pulse | | |  | | | |
|  |  |  | **Lesion** Ulcer □ Induration □ Scar/Epithelialising □ | | | | | | | | | | | |
| DATE | | **TIME** | | Multi-Disciplinary Notes | | | | | | | | **SIGNATURE** | | |
|  | |  | |  | | | | | | | |  | | |
|  | |  | |  | | | | | | | |  | | |
|  | |  | |  | | | | | | | |  | | |
|  | |  | |  | | | | | | | |  | | |
|  | |  | |  | | | | | | | |  | | |
|  | |  | |  | | | | | | | |  | | |
|  | |  | |  | | | | | | | |  | | |
|  | |  | |  | | | | | | | |  | | |
|  | |  | |  | | | | | | | |  | | |
|  | |  | |  | | | | | | | |  | | |
|  | |  | |  | | | | | | | |  | | |
|  | |  | |  | | | | | | | |  | | |

| 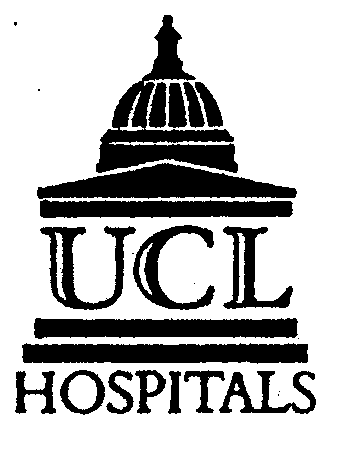  ***Assessment of Lesion***  ***Cutaneous Leishmaniasis***  Date: __________________________________ | | Attach Patient Identification Sticker or complete  **PATIENT NAME**  __________________________________________  HOSPITAL NUMBER __________________________________________ | |
| --- | --- | --- | --- |
| ***LOCATION OF LESION***  ***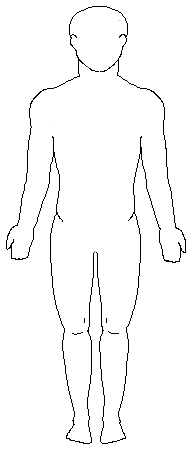*** | ***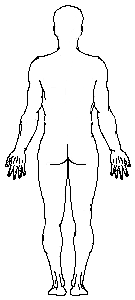*** | | ***DIAGRAM OF LESION***  (DIMENSIONS IN CM) |

***Initial assessment*** (Nurse to complete)

| **LESION GRADE (From lesion and pressure area care policy) 0 1 2 3 4** | **INFECTION** Swab sent **YES / NO** Date ________ |
| --- | --- |
| **LESION FLOOR CONDITION**  Healthy Granulation **YES / NO**  Thick Slough (Yellow / Brown) **YES / NO** | **RESULT**: Date:________ |
| Necrotic (Black) **YES /NO**  Cellulitis **YES / NO** | **ODOUR YES / NO** |
| **EXUDATE**  Colour  Amount | **PAIN** None  Dressing changes only |
| **CONDITION OF SURROUNDING SKIN**  e.g. Blisters, Fragile, etc. | Continuous  Name of analgesic: |

***Planning*  (Nurse to complete)**

| Debridement Method: |
| --- |
| Cleansing Solution: |
| Topical Agent / Dressing required: |
| Amount of dressing required per dressing change / size of dressing needed (Approx) |
| Frequency of dressing change per day and rationale (e.g. as per protocol) |
| LESION ASSESSED BY (Print & Sign) ________________________ GRADE ___________Review Date __________ |

**PHARMACY DRESSING SUPPLY** (Pharmacist to complete)

| **Dressing** | | | | | |  | | |  | | |  | | |  | | | |  | |
| --- | --- | --- | --- | --- | --- | --- | --- | --- | --- | --- | --- | --- | --- | --- | --- | --- | --- | --- | --- | --- |
| **Quantity supplied** | | | | | |  | | |  | | |  | | |  | | | |  | |
| **Date supplied** | | | | | |  | | |  | | |  | | |  | | | |  | |
| **Signature of Pharmacist** | | | | | |  | | |  | | |  | | |  | | | |  | |
| Day Case - Cut Leishmaniasis ICP | | | | | | | AFFIX PATIENT ID LABEL HERE | | | | | | Date | | | |  | | |  |
| NURSING | **Code** | | ACTION | | | | | Time | | **Sign** | **Reason for variance & action taken (use codes)** | | | | | | | **Sign** | |  |
|  | **Day 14** | | | | | | | | | | | | | | | | | | |  |
|  | **N104** | | Lesion redressed according to plan | | | | |  | |  |  | | | | | | |  | |  |
|  | **N105** | | IV sodium stibogluconate administered (ref. p. 105) | | | | |  | |  |  | | | | | | |  | |  |
|  | **N106** | | Patient observed for any adverse events (record below) | | | | |  | |  |  | | | | | | |  | |  |
|  | **N107** | | **Adverse Events** | | | | | | | **Action taken** | | | | | | | | | |  |
|  |  |  | Nausea: yes □ no □ | | | | | | |  | | | | | | | | | |  |
|  |  |  | Malaise: yes □ no □ | | | | | | |  | | | | | | | | | |  |
|  |  |  | Abdominal pain: yes □ no □ | | | | | | |  | | | | | | | | | |  |
|  |  |  | Myalgia: yes □ no □ | | | | | | |  | | | | | | | | | |  |
|  |  |  | Skin rash: yes □ no □  Location: | | | | | | |  | | | | | | | | | |  |
|  | **N108** | | SHO/SpR informed of adverse events | | | | |  | |  |  | | | | | | |  | |  |
|  | N109 | | Observations (before treatment) | | | | |  | |  |  | | | | | | |  | |  |
|  |  |  | Blood pressure | |  | | | | | Temperature | | | |  | | | | | |  |
|  |  |  | Respiration | |  | | | | | Pulse | | | |  | | | | | |  |
|  |  |  | Observations (during treatment) | | | | |  | |  |  | | | | | | |  | |  |
|  |  |  | Blood pressure | |  | | | | | Temperature | | | |  | | | | | |  |
|  |  |  | Respiration | |  | | | | | Pulse | | | |  | | | | | |  |
|  |  |  | Observations (after treatment) | | | | |  | |  |  | | | | | | |  | |  |
|  |  |  | Blood pressure | |  | | | | | Temperature | | | |  | | | | | |  |
|  |  |  | Respiration | |  | | | | | Pulse | | | |  | | | | | |  |
| **DATE** | | **TIME** | | Multi-Disciplinary Notes | | | | | | | | | | | | **SIGNATURE** | | | |  |
|  | |  | |  | | | | | | | | | | | |  | | | |  |
|  | |  | |  | | | | | | | | | | | |  | | | |  |
|  | |  | |  | | | | | | | | | | | |  | | | |  |
|  | |  | |  | | | | | | | | | | | |  | | | |  |
|  | |  | |  | | | | | | | | | | | |  | | | |  |
|  | |  | |  | | | | | | | | | | | |  | | | |  |
|  | |  | |  | | | | | | | | | | | |  | | | |  |
|  | |  | |  | | | | | | | | | | | |  | | | |  |
|  | |  | |  | | | | | | | | | | | |  | | | |  |
|  | |  | |  | | | | | | | | | | | |  | | | |  |
|  | |  | |  | | | | | | | | | | | |  | | | |  |
|  | |  | |  | | | | | | | | | | | |  | | | |  |
|  | |  | |  | | | | | | | | | | | |  | | | |  |
|  | |  | |  | | | | | | | | | | | |  | | | |  |
|  | |  | |  | | | | | | | | | | | |  | | | |  |
|  | |  | |  | | | | | | | | | | | |  | | | |  |
|  | |  | |  | | | | | | | | | | | |  | | | |  |
|  | |  | |  | | | | | | | | | | | |  | | | |  |

| Day Case - Cut Leishmaniasis ICP | | | | | | AFFIX PATIENT ID LABEL HERE | | | | Date | | |  | |
| --- | --- | --- | --- | --- | --- | --- | --- | --- | --- | --- | --- | --- | --- | --- |
| NURSING | **Code** | | ACTION | | | | Time | **Sign** | **Reason for variance & action taken (use codes)** | | | | | **Sign** |
|  | **Day 15** | | | | | | | | | | | | | |
|  | **N110** | | Lesion redressed according to plan | | | |  |  |  | | | | |  |
|  | **N111** | | IV sodium stibogluconate administered (ref. p. 105) | | | |  |  |  | | | | |  |
|  | **N112** | | Patient observed for any adverse events (record below) | | | |  |  |  | | | | |  |
|  | **N113** | | **Adverse Events** | | | | | **Action taken** | | | | | | |
|  |  |  | Nausea: yes □ no □ | | | | |  | | | | | | |
|  |  |  | Malaise: yes □ no □ | | | | |  | | | | | | |
|  |  |  | Abdominal pain: yes □ no □ | | | | |  | | | | | | |
|  |  |  | Myalgia: yes □ no □ | | | | |  | | | | | | |
|  |  |  | Skin rash: yes □ no □  Location: | | | | |  | | | | | | |
|  | **N114** | | SHO/SpR informed of adverse events | | | |  |  |  | | | | |  |
|  | N115 | | Observations (before treatment) | | | |  |  |  | | | | |  |
|  |  |  | Blood pressure | |  | | | Temperature | | |  | | | |
|  |  |  | Respiration | |  | | | Pulse | | |  | | | |
|  |  |  | Observations (during treatment) | | | |  |  |  | | | | |  |
|  |  |  | Blood pressure | |  | | | Temperature | | |  | | | |
|  |  |  | Respiration | |  | | | Pulse | | |  | | | |
|  |  |  | Observations (after treatment) | | | |  |  |  | | | | |  |
|  |  |  | Blood pressure | |  | | | Temperature | | |  | | | |
|  |  |  | Respiration | |  | | | Pulse | | |  | | | |
| **DATE** | | **TIME** | | Multi-Disciplinary Notes | | | | | | | | **SIGNATURE** | | |
|  | |  | |  | | | | | | | |  | | |
|  | |  | |  | | | | | | | |  | | |
|  | |  | |  | | | | | | | |  | | |
|  | |  | |  | | | | | | | |  | | |
|  | |  | |  | | | | | | | |  | | |
|  | |  | |  | | | | | | | |  | | |
|  | |  | |  | | | | | | | |  | | |
|  | |  | |  | | | | | | | |  | | |
|  | |  | |  | | | | | | | |  | | |
|  | |  | |  | | | | | | | |  | | |
|  | |  | |  | | | | | | | |  | | |
|  | |  | |  | | | | | | | |  | | |
|  | |  | |  | | | | | | | |  | | |
|  | |  | |  | | | | | | | |  | | |
|  | |  | |  | | | | | | | |  | | |
|  | |  | |  | | | | | | | |  | | |
|  | |  | |  | | | | | | | |  | | |

| Day Case - Cut Leishmaniasis ICP | | | | | | AFFIX PATIENT ID LABEL HERE | | | | Date | | |  | |
| --- | --- | --- | --- | --- | --- | --- | --- | --- | --- | --- | --- | --- | --- | --- |
| NURSING | **Code** | | ACTION | | | | Time | **Sign** | **Reason for variance & action taken (use codes)** | | | | | **Sign** |
|  | **Day 16** | | | | | | | | | | | | | |
|  | **N116** | | Lesion redressed according to plan | | | |  |  |  | | | | |  |
|  | N117 | | FBC taken and sent | | | |  |  |  | | | | |  |
|  | N118 | | LFT taken and sent | | | |  |  |  | | | | |  |
|  | N119 | | U & E taken and sent | | | |  |  |  | | | | |  |
|  | N120 | | ECG recorded | | | |  |  |  | | | | |  |
|  | **N121** | | IV sodium stibogluconate administered (ref. p. 101) | | | |  |  |  | | | | |  |
|  | **N122** | | Patient observed for any adverse events (record below) | | | |  |  |  | | | | |  |
|  | **N123** | | **Adverse Events** | | | | | **Action taken** | | | | | | |
|  |  |  | Nausea: yes □ no □ | | | | |  | | | | | | |
|  |  |  | Malaise: yes □ no □ | | | | |  | | | | | | |
|  |  |  | Abdominal pain: yes □ no □ | | | | |  | | | | | | |
|  |  |  | Myalgia: yes □ no □ | | | | |  | | | | | | |
|  |  |  | Skin rash: yes □ no □  Location: | | | | |  | | | | | | |
|  | **N124** | | Patient reviewed by SHO or SpR | | | |  |  |  | | | | |  |
|  | **N125** | | Observations (before treatment) | | | |  |  |  | | | | |  |
|  |  |  | Blood pressure | |  | | | Temperature | | |  | | | |
|  |  |  | Respiration | |  | | | Pulse | | |  | | | |
|  |  |  | Observations (during treatment) | | | |  |  |  | | | | |  |
|  |  |  | Blood pressure | |  | | | Temperature | | |  | | | |
|  |  |  | Respiration | |  | | | Pulse | | |  | | | |
|  |  |  | Observations (after treatment) | | | |  |  |  | | | | |  |
|  |  |  | Blood pressure | |  | | | Temperature | | |  | | | |
|  |  |  | Respiration | |  | | | Pulse | | |  | | | |
|  |  |  | **Lesion** Ulcer □ Induration □ Scar/Epithelialising □ | | | | | | | | | | | |
| **DATE** | | **TIME** | | Multi-Disciplinary Notes | | | | | | | | **SIGNATURE** | | |
|  | |  | |  | | | | | | | |  | | |
|  | |  | |  | | | | | | | |  | | |
|  | |  | |  | | | | | | | |  | | |
|  | |  | |  | | | | | | | |  | | |
|  | |  | |  | | | | | | | |  | | |
|  | |  | |  | | | | | | | |  | | |
|  | |  | |  | | | | | | | |  | | |
|  | |  | |  | | | | | | | |  | | |
|  | |  | |  | | | | | | | |  | | |
|  | |  | |  | | | | | | | |  | | |

| Day Case - Cut Leishmaniasis ICP | | | | | | AFFIX PATIENT ID LABEL HERE | | | | Date | | |  | |
| --- | --- | --- | --- | --- | --- | --- | --- | --- | --- | --- | --- | --- | --- | --- |
| NURSING | **Code** | | ACTION | | | | Time | **Sign** | **Reason for variance & action taken (use codes)** | | | | | **Sign** |
|  | **Day 17** | | | | | | | | | | | | | |
|  | N126 | | Lesion redressed according to plan | | | |  |  |  | | | | |  |
|  | **N127** | | IV sodium stibogluconate administered (ref. p. 105) | | | |  |  |  | | | | |  |
|  | **N128** | | Patient observed for any adverse events (record below) | | | |  |  |  | | | | |  |
|  | **N129** | | **Adverse Events** | | | | | **Action taken** | | | | | | |
|  |  |  | Nausea: yes □ no □ | | | | |  | | | | | | |
|  |  |  | Malaise: yes □ no □ | | | | |  | | | | | | |
|  |  |  | Abdominal pain: yes □ no □ | | | | |  | | | | | | |
|  |  |  | Myalgia: yes □ no □ | | | | |  | | | | | | |
|  |  |  | Skin rash: yes □ no □  Location: | | | | |  | | | | | | |
|  | **N130** | | SHO/SpR informed of adverse events | | | |  |  |  | | | | |  |
|  |  |  | Observations (before treatment) | | | |  |  |  | | | | |  |
|  |  |  | Blood pressure | |  | | | Temperature | | |  | | | |
|  |  |  | Respiration | |  | | | Pulse | | |  | | | |
|  |  |  | Observations (during treatment) | | | |  |  |  | | | | |  |
|  |  |  | Blood pressure | |  | | | Temperature | | |  | | | |
|  |  |  | Respiration | |  | | | Pulse | | |  | | | |
|  |  |  | Observations (after treatment) | | | |  |  |  | | | | |  |
|  |  |  | Blood pressure | |  | | | Temperature | | |  | | | |
|  |  |  | Respiration | |  | | | Pulse | | |  | | | |
| **DATE** | | **TIME** | | Multi-Disciplinary Notes | | | | | | | | **SIGNATURE** | | |
|  | |  | |  | | | | | | | |  | | |
|  | |  | |  | | | | | | | |  | | |
|  | |  | |  | | | | | | | |  | | |
|  | |  | |  | | | | | | | |  | | |
|  | |  | |  | | | | | | | |  | | |
|  | |  | |  | | | | | | | |  | | |
|  | |  | |  | | | | | | | |  | | |
|  | |  | |  | | | | | | | |  | | |
|  | |  | |  | | | | | | | |  | | |
|  | |  | |  | | | | | | | |  | | |
|  | |  | |  | | | | | | | |  | | |
|  | |  | |  | | | | | | | |  | | |
|  | |  | |  | | | | | | | |  | | |
|  | |  | |  | | | | | | | |  | | |
|  | |  | |  | | | | | | | |  | | |

| Day Case - Cut Leishmaniasis ICP | | | | | | AFFIX PATIENT ID LABEL HERE | | | | Date | | |  | |
| --- | --- | --- | --- | --- | --- | --- | --- | --- | --- | --- | --- | --- | --- | --- |
| NURSING | **Code** | | ACTION | | | | Time | **Sign** | **Reason for variance & action taken (use codes)** | | | | | **Sign** |
|  | **Day 18** | | | | | | | | | | | | | |
|  | N131 | | Lesion redressed according to plan | | | |  |  |  | | | | |  |
|  | **N132** | | IV sodium stibogluconate administered (ref. p. 105) | | | |  |  |  | | | | |  |
|  | **N133** | | Patient observed for any adverse events (record below) | | | |  |  |  | | | | |  |
|  | **N134** | | **Adverse Events** | | | | | **Action taken** | | | | | | |
|  |  |  | Nausea: yes □ no □ | | | | |  | | | | | | |
|  |  |  | Malaise: yes □ no □ | | | | |  | | | | | | |
|  |  |  | Abdominal pain: yes □ no □ | | | | |  | | | | | | |
|  |  |  | Myalgia: yes □ no □ | | | | |  | | | | | | |
|  |  |  | Skin rash: yes □ no □  Location: | | | | |  | | | | | | |
|  | **N135** | | SHO/SpR informed of adverse events | | | |  |  |  | | | | |  |
|  |  |  | Observations (before treatment) | | | |  |  |  | | | | |  |
|  |  |  | Blood pressure | |  | | | Temperature | | |  | | | |
|  |  |  | Respiration | |  | | | Pulse | | |  | | | |
|  |  |  | Observations (during treatment) | | | |  |  |  | | | | |  |
|  |  |  | Blood pressure | |  | | | Temperature | | |  | | | |
|  |  |  | Respiration | |  | | | Pulse | | |  | | | |
|  |  |  | Observations (after treatment) | | | |  |  |  | | | | |  |
|  |  |  | Blood pressure | |  | | | Temperature | | |  | | | |
|  |  |  | Respiration | |  | | | Pulse | | |  | | | |
| **DATE** | | **TIME** | | Multi-Disciplinary Notes | | | | | | | | **SIGNATURE** | | |
|  | |  | |  | | | | | | | |  | | |
|  | |  | |  | | | | | | | |  | | |
|  | |  | |  | | | | | | | |  | | |
|  | |  | |  | | | | | | | |  | | |
|  | |  | |  | | | | | | | |  | | |
|  | |  | |  | | | | | | | |  | | |
|  | |  | |  | | | | | | | |  | | |
|  | |  | |  | | | | | | | |  | | |
|  | |  | |  | | | | | | | |  | | |
|  | |  | |  | | | | | | | |  | | |
|  | |  | |  | | | | | | | |  | | |
|  | |  | |  | | | | | | | |  | | |
|  | |  | |  | | | | | | | |  | | |
|  | |  | |  | | | | | | | |  | | |
|  | |  | |  | | | | | | | |  | | |
|  | |  | |  | | | | | | | |  | | |
|  | |  | |  | | | | | | | |  | | |

| Day Case - Cut Leishmaniasis ICP | | | | | | AFFIX PATIENT ID LABEL HERE | | | | Date | | |  | |
| --- | --- | --- | --- | --- | --- | --- | --- | --- | --- | --- | --- | --- | --- | --- |
| NURSING | **Code** | | ACTION | | | | Time | **Sign** | **Reason for variance & action taken (use codes)** | | | | | **Sign** |
|  | **Day 19** | | | | | | | | | | | | | |
|  | **N136** | | Lesion redressed according to plan | | | |  |  |  | | | | |  |
|  | N137 | | FBC taken and sent | | | |  |  |  | | | | |  |
|  | N138 | | LFT taken and sent | | | |  |  |  | | | | |  |
|  | N139 | | U & E taken and sent | | | |  |  |  | | | | |  |
|  | N140 | | ECG recorded | | | |  |  |  | | | | |  |
|  | **N141** | | IV sodium stibogluconate administered (ref. p. 101) | | | |  |  |  | | | | |  |
|  | **N142** | | Patient observed for any adverse events (record below) | | | |  |  |  | | | | |  |
|  | **N143** | | **Adverse Events** | | | | | **Action taken** | | | | | | |
|  |  |  | Nausea: yes □ no □ | | | | |  | | | | | | |
|  |  |  | Malaise: yes □ no □ | | | | |  | | | | | | |
|  |  |  | Abdominal pain: yes □ no □ | | | | |  | | | | | | |
|  |  |  | Myalgia: yes □ no □ | | | | |  | | | | | | |
|  |  |  | Skin rash: yes □ no □  Location: | | | | |  | | | | | | |
|  | **N144** | | Patient reviewed by SHO or SpR | | | |  |  |  | | | | |  |
|  | N145 | | Observations (before treatment) | | | |  |  |  | | | | |  |
|  |  |  | Blood pressure | |  | | | Temperature | | |  | | | |
|  |  |  | Respiration | |  | | | Pulse | | |  | | | |
|  |  |  | Observations (during treatment) | | | |  |  |  | | | | |  |
|  |  |  | Blood pressure | |  | | | Temperature | | |  | | | |
|  |  |  | Respiration | |  | | | Pulse | | |  | | | |
|  |  |  | Observations (after treatment) | | | |  |  |  | | | | |  |
|  |  |  | Blood pressure | |  | | | Temperature | | |  | | | |
|  |  |  | Respiration | |  | | | Pulse | | |  | | | |
|  |  |  | **Lesion** Ulcer □ Induration □ Scar/Epithelialising □ | | | | | | | | | | | |
| **DATE** | | **TIME** | | Multi-Disciplinary Notes | | | | | | | | **SIGNATURE** | | |
|  | |  | |  | | | | | | | |  | | |
|  | |  | |  | | | | | | | |  | | |
|  | |  | |  | | | | | | | |  | | |
|  | |  | |  | | | | | | | |  | | |
|  | |  | |  | | | | | | | |  | | |
|  | |  | |  | | | | | | | |  | | |
|  | |  | |  | | | | | | | |  | | |
|  | |  | |  | | | | | | | |  | | |
|  | |  | |  | | | | | | | |  | | |
|  | |  | |  | | | | | | | |  | | |
|  | |  | |  | | | | | | | |  | | |

| Day Case - Cut Leishmaniasis ICP | | | | | | AFFIX PATIENT ID LABEL HERE | | | | Date | | |  | |
| --- | --- | --- | --- | --- | --- | --- | --- | --- | --- | --- | --- | --- | --- | --- |
| NURSING | **Code** | | ACTION | | | | Time | **Sign** | **Reason for variance & action taken (use codes)** | | | | | **Sign** |
|  | **Day 20** | | | | | | | | | | | | | |
|  | N146 | | Lesion redressed according to plan | | | |  |  |  | | | | |  |
|  | **N147** | | IV sodium stibogluconate administered (ref p. 105) | | | |  |  |  | | | | |  |
|  | **N148** | | Patient observed for any adverse events (record below) | | | |  |  |  | | | | |  |
|  | **N149** | | **Adverse Events** | | | | | **Action taken** | | | | | | |
|  |  |  | Nausea: yes □ no □ | | | | |  | | | | | | |
|  |  |  | Malaise: yes □ no □ | | | | |  | | | | | | |
|  |  |  | Abdominal pain: yes □ no □ | | | | |  | | | | | | |
|  |  |  | Myalgia: yes □ no □ | | | | |  | | | | | | |
|  |  |  | Skin rash: yes □ no □  Location: | | | | |  | | | | | | |
|  | **N150** | | SHO/SpR informed of adverse events | | | |  |  |  | | | | |  |
|  |  |  | Observations (before treatment) | | | |  |  |  | | | | |  |
|  |  |  | Blood pressure | |  | | | Temperature | | |  | | | |
|  |  |  | Respiration | |  | | | Pulse | | |  | | | |
|  |  |  | Observations (during treatment) | | | |  |  |  | | | | |  |
|  |  |  | Blood pressure | |  | | | Temperature | | |  | | | |
|  |  |  | Respiration | |  | | | Pulse | | |  | | | |
|  |  |  | Observations (after treatment) | | | |  |  |  | | | | |  |
|  |  |  | Blood pressure | |  | | | Temperature | | |  | | | |
|  |  |  | Respiration | |  | | | Pulse | | |  | | | |
| **DATE** | | **TIME** | | Multi-Disciplinary Notes | | | | | | | | **SIGNATURE** | | |
|  | |  | |  | | | | | | | |  | | |
|  | |  | |  | | | | | | | |  | | |
|  | |  | |  | | | | | | | |  | | |
|  | |  | |  | | | | | | | |  | | |
|  | |  | |  | | | | | | | |  | | |
|  | |  | |  | | | | | | | |  | | |
|  | |  | |  | | | | | | | |  | | |
|  | |  | |  | | | | | | | |  | | |
|  | |  | |  | | | | | | | |  | | |
|  | |  | |  | | | | | | | |  | | |
|  | |  | |  | | | | | | | |  | | |
|  | |  | |  | | | | | | | |  | | |
|  | |  | |  | | | | | | | |  | | |
|  | |  | |  | | | | | | | |  | | |
|  | |  | |  | | | | | | | |  | | |
|  | |  | |  | | | | | | | |  | | |

| Day Case - Cut Leishmaniasis ICP | | | | AFFIX PATIENT ID LABEL HERE | | | | Date | |  | |
| --- | --- | --- | --- | --- | --- | --- | --- | --- | --- | --- | --- |
| NURSING | **Code** | ACTION | | | Time | **Sign** | **Reason for variance & action taken (use codes)** | | | | **Sign** |
|  | **Day 21** | | | | | | | | | | |
|  | N151 | Lesion reassessment chart completed (P51) | | |  |  |  | | | |  |
|  | **N152** | Lesion redressed according to reassessment (P51) | | |  |  |  | | | |  |
|  | N153 | FBC taken and sent | | |  |  |  | | | |  |
|  | N154 | LFT taken and sent | | |  |  |  | | | |  |
|  | N155 | U & E taken and sent | | |  |  |  | | | |  |
|  | N156 | ECG recorded | | |  |  |  | | | |  |
|  | **N157** | IV sodium stibogluconate administered (ref. p. 105) | | |  |  |  | | | |  |
|  | **N158** | Patient observed for any adverse events (record below) | | |  |  |  | | | |  |
|  | **N159** | **Adverse Events** | | | | **Action taken** | | | | | |
|  |  | Nausea: yes □ no □ | | | |  | | | | | |
|  |  | Malaise: yes □ no □ | | | |  | | | | | |
|  |  | Abdominal pain: yes □ no □ | | | |  | | | | | |
|  |  | Myalgia: yes □ no □ | | | |  | | | | | |
|  |  | Skin rash: yes □ no □  Location: | | | |  | | | | | |
|  | **N160** | Patient reviewed by SHO or SpR | | |  |  |  | | | |  |
|  | N161 | Observations (before treatment) | | |  |  |  | | | |  |
|  |  | Blood pressure |  | | | Temperature | | |  | | |
|  |  | Respiration |  | | | Pulse | | |  | | |
|  |  | Observations (during treatment) | | |  |  |  | | | |  |
|  |  | Blood pressure |  | | | Temperature | | |  | | |
|  |  | Respiration |  | | | Pulse | | |  | | |
|  |  | Observations (after treatment) | | |  |  |  | | | |  |
|  |  | Blood pressure |  | | | Temperature | | |  | | |
|  |  | Respiration |  | | | Pulse | | |  | | |
|  |  | **Lesion** Ulcer □ Induration □ Scar/Epithelialising □ | | | | | | | | | |

| Day Case - Cut Leishmaniasis ICP | | | | | | AFFIX PATIENT ID LABEL HERE | | | | | | | Date | |  | |  |
| --- | --- | --- | --- | --- | --- | --- | --- | --- | --- | --- | --- | --- | --- | --- | --- | --- | --- |
| NURSING | **Code** | | ACTION | | | | Time | **Sign** | **Reason for variance & action taken (use codes)** | | | | | | | **Sign** |  |
|  | **Day 21 (cont’d)** | | | | | | | | | | | | | | | |  |
|  | **N162** | | **Discharge**   - Patient has been reviewed by SHO/SpR - Confirm clinically fit for discharge - Patient understands s/he is to contact clinic if unwell in the following few days - Patient taught care of lesion - Patient informed how to obtain dressings when required - Outpatients appointment booked - Patient has no complaints - Discharged from electronic system - If hotel stay patient –ensure hotel aware booking now ended | | | |  |  | | | |  | | | |  |  |
| **DATE** | | **TIME** | | Multi-Disciplinary Notes | | | | | | | | | | **SIGNATURE** | | |  |
|  | |  | |  | | | | | | | | | |  | | |  |
|  | |  | |  | | | | | | | | | |  | | |  |
|  | |  | |  | | | | | | | | | |  | | |  |
|  | |  | |  | | | | | | | | | |  | | |  |
|  | |  | |  | | | | | | | | | |  | | |  |
|  | |  | |  | | | | | | | | | |  | | |  |
|  | |  | |  | | | | | | | | | |  | | |  |
|  | |  | |  | | | | | | | | | |  | | |  |
|  | |  | |  | | | | | | | | | |  | | |  |
|  | |  | |  | | | | | | | | | |  | | |  |
|  | |  | |  | | | | | | | | | |  | | |  |
|  | |  | |  | | | | | | | | | |  | | |  |
|  | |  | |  | | | | | | | | | |  | | |  |
|  | |  | |  | | | | | | | | | |  | | |  |
|  | |  | |  | | | | | | | | | |  | | |  |
|  | |  | |  | | | | | | | | | |  | | |  |
|  | |  | |  | | | | | | | | | |  | | |  |
|  | |  | |  | | | | | | | | | |  | | |  |
|  | |  | |  | | | | | | | | | |  | | |  |
|  | |  | |  | | | | | | | | | |  | | |  |
|  | |  | |  | | | | | | | | | |  | | |  |
|  | |  | |  | | | | | | | | | |  | | |  |
|  | |  | |  | | | | | | | | | |  | | |  |
|  | |  | |  | | | | | | | | | |  | | |  |
|  | |  | |  | | | | | | | | | |  | | |  |
|  | |  | |  | | | | | | | | | |  | | |  |
|  | |  | |  | | | | | | | | | |  | | |  |
|  | |  | |  | | | | | | | | | |  | | |  |
|  | |  | |  | | | | | | | | | |  | | |  |
|  | |  | |  | | | | | | | | | |  | | |  |
|  | |  | |  | | | | | | | | | |  | | |  |
| 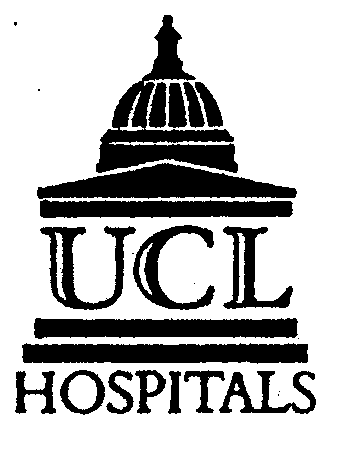  ***Assessment of Lesion***  ***Cutaneous Leishmaniasis***  Date: __________________________________ | | | | | | | | | | Attach Patient Identification Sticker or complete  **PATIENT NAME**  __________________________________________  HOSPITAL NUMBER __________________________________________ | | | | | | | |
| ***LOCATION OF LESION***  ***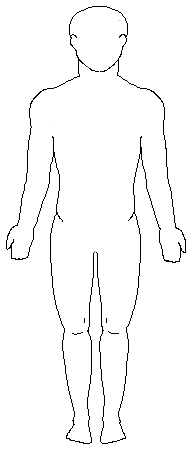*** | | | | | ***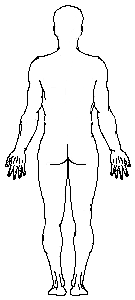*** | | | | | | ***DIAGRAM OF LESION***  (DIMENSIONS IN CM) | | | | | | |

| **LESION GRADE (From lesion and pressure area care policy) 0 1 2 3 4** | **INFECTION** Swab sent **YES / NO** Date ________ |
| --- | --- |
| **LESION FLOOR CONDITION**  Healthy Granulation **YES / NO**  Thick Slough (Yellow / Brown) **YES / NO** | **RESULT**: Date:________ |
| Necrotic (Black) **YES /NO**  Cellulitis **YES / NO** | **ODOUR YES / NO** |
| **EXUDATE**  Colour  Amount | **PAIN** None  Dressing changes only |
| **CONDITION OF SURROUNDING SKIN**  e.g. Blisters, Fragile, etc. | Continuous  Name of analgesic: |

***Planning*  (Nurse to complete)**

| Debridement Method: |
| --- |
| Cleansing Solution: |
| Topical Agent / Dressing required: |
| Amount of dressing required per dressing change / size of dressing needed (Approx) |
| Frequency of dressing change per day and rationale (e.g. as per protocol) |
| LESION ASSESSED BY (Print & Sign) ________________________ GRADE ___________Review Date __________ |

**PHARMACY DRESSING SUPPLY (Pharmacist to complete)**

| **Dressing** |  |  |  |  |  |
| --- | --- | --- | --- | --- | --- |
| **Quantity supplied** |  |  |  |  |  |
| **Date supplied** |  |  |  |  |  |
| **Signature of Pharmacist** |  |  |  |  |  |

| Day Case - Cut Leishmaniasis ICP | | | | | | AFFIX PATIENT ID LABEL HERE | | | | Date | | |  | |
| --- | --- | --- | --- | --- | --- | --- | --- | --- | --- | --- | --- | --- | --- | --- |
| NURSING | **Code** | | ACTION | | | | Time | **Sign** | **Reason for variance & action taken (use codes)** | | | | | **Sign** |
|  | **Day 22** | | | | | | | | | | | | | |
|  | **N163** | | Lesion redressed according to plan | | | |  |  |  | | | | |  |
|  | **N164** | | IV sodium stibogluconate administered (ref. p. 105) | | | |  |  |  | | | | |  |
|  | **N165** | | Patient observed for any adverse events (record below) | | | |  |  |  | | | | |  |
|  | **N166** | | **Adverse Events** | | | | | **Action taken** | | | | | | |
|  |  |  | Nausea: yes □ no □ | | | | |  | | | | | | |
|  |  |  | Malaise: yes □ no □ | | | | |  | | | | | | |
|  |  |  | Abdominal pain: yes □ no □ | | | | |  | | | | | | |
|  |  |  | Myalgia: yes □ no □ | | | | |  | | | | | | |
|  |  |  | Skin rash: yes □ no □  Location: | | | | |  | | | | | | |
|  | **N167** | | SHO/SpR informed of adverse events | | | |  |  |  | | | | |  |
|  | **N168** | | Observations (before treatment) | | | |  |  |  | | | | |  |
|  |  |  | Blood pressure | |  | | | Temperature | | |  | | | |
|  |  |  | Respiration | |  | | | Pulse | | |  | | | |
|  |  |  | Observations (during treatment) | | | |  |  |  | | | | |  |
|  |  |  | Blood pressure | |  | | | Temperature | | |  | | | |
|  |  |  | Respiration | |  | | | Pulse | | |  | | | |
|  |  |  | Observations (after treatment) | | | |  |  |  | | | | |  |
|  |  |  | Blood pressure | |  | | | Temperature | | |  | | | |
|  |  |  | Respiration | |  | | | Pulse | | |  | | | |
| **DATE** | | **TIME** | | Multi-Disciplinary Notes | | | | | | | | **SIGNATURE** | | |
|  | |  | |  | | | | | | | |  | | |
|  | |  | |  | | | | | | | |  | | |
|  | |  | |  | | | | | | | |  | | |
|  | |  | |  | | | | | | | |  | | |
|  | |  | |  | | | | | | | |  | | |
|  | |  | |  | | | | | | | |  | | |
|  | |  | |  | | | | | | | |  | | |
|  | |  | |  | | | | | | | |  | | |
|  | |  | |  | | | | | | | |  | | |
|  | |  | |  | | | | | | | |  | | |
|  | |  | |  | | | | | | | |  | | |
|  | |  | |  | | | | | | | |  | | |
|  | |  | |  | | | | | | | |  | | |
|  | |  | |  | | | | | | | |  | | |
|  | |  | |  | | | | | | | |  | | |
|  | |  | |  | | | | | | | |  | | |

| Day Case - Cut Leishmaniasis ICP | | | | | | AFFIX PATIENT ID LABEL HERE | | | | Date | | |  | |
| --- | --- | --- | --- | --- | --- | --- | --- | --- | --- | --- | --- | --- | --- | --- |
| NURSING | **Code** | | ACTION | | | | Time | **Sign** | **Reason for variance & action taken (use codes)** | | | | | **Sign** |
|  | **Day 23** | | | | | | | | | | | | | |
|  | N169 | | Lesion redressed according to plan | | | |  |  |  | | | | |  |
|  | **N170** | | IV sodium stibogluconate administered (ref. p. 105) | | | |  |  |  | | | | |  |
|  | **N171** | | Patient observed for any adverse events (record below) | | | |  |  |  | | | | |  |
|  | **N172** | | **Adverse Events** | | | | | **Action taken** | | | | | | |
|  |  |  | Nausea: yes □ no □ | | | | |  | | | | | | |
|  |  |  | Malaise: yes □ no □ | | | | |  | | | | | | |
|  |  |  | Abdominal pain: yes □ no □ | | | | |  | | | | | | |
|  |  |  | Myalgia: yes □ no □ | | | | |  | | | | | | |
|  |  |  | Skin rash: yes □ no □  Location: | | | | |  | | | | | | |
|  | **N173** | | SHO/SpR informed of adverse events | | | |  |  |  | | | | |  |
|  | **N174** | | Observations (before treatment) | | | |  |  |  | | | | |  |
|  |  |  | Blood pressure | |  | | | Temperature | | |  | | | |
|  |  |  | Respiration | |  | | | Pulse | | |  | | | |
|  |  |  | Observations (during treatment) | | | |  |  |  | | | | |  |
|  |  |  | Blood pressure | |  | | | Temperature | | |  | | | |
|  |  |  | Respiration | |  | | | Pulse | | |  | | | |
|  |  |  | Observations (after treatment) | | | |  |  |  | | | | |  |
|  |  |  | Blood pressure | |  | | | Temperature | | |  | | | |
|  |  |  | Respiration | |  | | | Pulse | | |  | | | |
| **DATE** | | **TIME** | | Multi-Disciplinary Notes | | | | | | | | **SIGNATURE** | | |
|  | |  | |  | | | | | | | |  | | |
|  | |  | |  | | | | | | | |  | | |
|  | |  | |  | | | | | | | |  | | |
|  | |  | |  | | | | | | | |  | | |
|  | |  | |  | | | | | | | |  | | |
|  | |  | |  | | | | | | | |  | | |
|  | |  | |  | | | | | | | |  | | |
|  | |  | |  | | | | | | | |  | | |
|  | |  | |  | | | | | | | |  | | |
|  | |  | |  | | | | | | | |  | | |
|  | |  | |  | | | | | | | |  | | |
|  | |  | |  | | | | | | | |  | | |
|  | |  | |  | | | | | | | |  | | |
|  | |  | |  | | | | | | | |  | | |
|  | |  | |  | | | | | | | |  | | |
|  | |  | |  | | | | | | | |  | | |
|  | |  | |  | | | | | | | |  | | |
|  | |  | |  | | | | | | | |  | | |
|  | |  | |  | | | | | | | |  | | |

| Day Case - Cut Leishmaniasis ICP | | | | | | AFFIX PATIENT ID LABEL HERE | | | | Date | | |  | |
| --- | --- | --- | --- | --- | --- | --- | --- | --- | --- | --- | --- | --- | --- | --- |
| NURSING | **Code** | | ACTION | | | | Time | **Sign** | **Reason for variance & action taken (use codes)** | | | | | **Sign** |
|  | **Day 24** | | | | | | | | | | | | | |
|  | N175 | | Lesion redressed according to plan | | | |  |  |  | | | | |  |
|  | N176 | | FBC taken and sent | | | |  |  |  | | | | |  |
|  | N177 | | LFT taken and sent | | | |  |  |  | | | | |  |
|  | N178 | | U & E taken and sent | | | |  |  |  | | | | |  |
|  | N179 | | ECG recorded | | | |  |  |  | | | | |  |
|  | **N180** | | IV sodium stibogluconate administered (ref. p. 105) | | | |  |  |  | | | | |  |
|  | **N181** | | Patient observed for any adverse events (record below) | | | |  |  |  | | | | |  |
|  | **N182** | | **Adverse Events** | | | | | **Action taken** | | | | | | |
|  |  |  | Nausea: yes □ no □ | | | | |  | | | | | | |
|  |  |  | Malaise: yes □ no □ | | | | |  | | | | | | |
|  |  |  | Abdominal pain: yes □ no □ | | | | |  | | | | | | |
|  |  |  | Myalgia: yes □ no □ | | | | |  | | | | | | |
|  |  |  | Skin rash: yes □ no □  Location: | | | | |  | | | | | | |
|  | **N183** | | Patient reviewed by SHO or SpR | | | |  |  |  | | | | |  |
|  | N184 | | Observations (before treatment) | | | |  |  |  | | | | |  |
|  |  |  | Blood pressure | |  | | | Temperature | | |  | | | |
|  |  |  | Respiration | |  | | | Pulse | | |  | | | |
|  |  |  | Observations (during treatment) | | | |  |  |  | | | | |  |
|  |  |  | Blood pressure | |  | | | Temperature | | |  | | | |
|  |  |  | Respiration | |  | | | Pulse | | |  | | | |
|  |  |  | Observations (after treatment) | | | |  |  |  | | | | |  |
|  |  |  | Blood pressure | |  | | | Temperature | | |  | | | |
|  |  |  | Respiration | |  | | | Pulse | | |  | | | |
|  |  |  | **Lesion** Ulcer □ Induration □ Scar/Epithelialising □ | | | | | | | | | | | |
| **DATE** | | **TIME** | | Multi-Disciplinary Notes | | | | | | | | **SIGNATURE** | | |
|  | |  | |  | | | | | | | |  | | |
|  | |  | |  | | | | | | | |  | | |
|  | |  | |  | | | | | | | |  | | |
|  | |  | |  | | | | | | | |  | | |
|  | |  | |  | | | | | | | |  | | |
|  | |  | |  | | | | | | | |  | | |
|  | |  | |  | | | | | | | |  | | |
|  | |  | |  | | | | | | | |  | | |
|  | |  | |  | | | | | | | |  | | |
|  | |  | |  | | | | | | | |  | | |
|  | |  | |  | | | | | | | |  | | |
|  | |  | |  | | | | | | | |  | | |

| Day Case - Cut Leishmaniasis ICP | | | | | | AFFIX PATIENT ID LABEL HERE | | | | Date | | |  | |
| --- | --- | --- | --- | --- | --- | --- | --- | --- | --- | --- | --- | --- | --- | --- |
| NURSING | **Code** | | ACTION | | | | Time | **Sign** | **Reason for variance & action taken (use codes)** | | | | | **Sign** |
|  | **Day 25** | | | | | | | | | | | | | |
|  | N185 | | Lesion redressed according to plan | | | |  |  |  | | | | |  |
|  | **N186** | | IV sodium stibogluconate administered (ref. p. 105) | | | |  |  |  | | | | |  |
|  | **N187** | | Patient observed for any adverse events (record below) | | | |  |  |  | | | | |  |
|  | **N188** | | **Adverse Events** | | | | | **Action taken** | | | | | | |
|  |  |  | Nausea: yes □ no □ | | | | |  | | | | | | |
|  |  |  | Malaise: yes □ no □ | | | | |  | | | | | | |
|  |  |  | Abdominal pain: yes □ no □ | | | | |  | | | | | | |
|  |  |  | Myalgia: yes □ no □ | | | | |  | | | | | | |
|  |  |  | Skin rash: yes □ no □  Location: | | | | |  | | | | | | |
|  | **N189** | | SHO/SpR informed of adverse events | | | |  |  |  | | | | |  |
|  | **N190** | | Observations (before treatment) | | | |  |  |  | | | | |  |
|  |  |  | Blood pressure | |  | | | Temperature | | |  | | | |
|  |  |  | Respiration | |  | | | Pulse | | |  | | | |
|  |  |  | Observations (during treatment) | | | |  |  |  | | | | |  |
|  |  |  | Blood pressure | |  | | | Temperature | | |  | | | |
|  |  |  | Respiration | |  | | | Pulse | | |  | | | |
|  |  |  | Observations (after treatment) | | | |  |  |  | | | | |  |
|  |  |  | Blood pressure | |  | | | Temperature | | |  | | | |
|  |  |  | Respiration | |  | | | Pulse | | |  | | | |
| **DATE** | | **TIME** | | Multi-Disciplinary Notes | | | | | | | | **SIGNATURE** | | |
|  | |  | |  | | | | | | | |  | | |
|  | |  | |  | | | | | | | |  | | |
|  | |  | |  | | | | | | | |  | | |
|  | |  | |  | | | | | | | |  | | |
|  | |  | |  | | | | | | | |  | | |
|  | |  | |  | | | | | | | |  | | |
|  | |  | |  | | | | | | | |  | | |
|  | |  | |  | | | | | | | |  | | |
|  | |  | |  | | | | | | | |  | | |
|  | |  | |  | | | | | | | |  | | |
|  | |  | |  | | | | | | | |  | | |
|  | |  | |  | | | | | | | |  | | |
|  | |  | |  | | | | | | | |  | | |
|  | |  | |  | | | | | | | |  | | |
|  | |  | |  | | | | | | | |  | | |
|  | |  | |  | | | | | | | |  | | |
|  | |  | |  | | | | | | | |  | | |

| Day Case - Cut Leishmaniasis ICP | | | | | | AFFIX PATIENT ID LABEL HERE | | | | Date | | |  | |
| --- | --- | --- | --- | --- | --- | --- | --- | --- | --- | --- | --- | --- | --- | --- |
| NURSING | **Code** | | ACTION | | | | Time | **Sign** | **Reason for variance & action taken (use codes)** | | | | | **Sign** |
|  | **Day 26** | | | | | | | | | | | | | |
|  | **N191** | | Lesion redressed according to plan | | | |  |  |  | | | | |  |
|  | **N192** | | IV sodium stibogluconate administered (ref. p. 105) | | | |  |  |  | | | | |  |
|  | **N192** | | Patient observed for any adverse events (record below) | | | |  |  |  | | | | |  |
|  | **N194** | | **Adverse Events** | | | | | **Action taken** | | | | | | |
|  |  |  | Nausea: yes □ no □ | | | | |  | | | | | | |
|  |  |  | Malaise: yes □ no □ | | | | |  | | | | | | |
|  |  |  | Abdominal pain: yes □ no □ | | | | |  | | | | | | |
|  |  |  | Myalgia: yes □ no □ | | | | |  | | | | | | |
|  |  |  | Skin rash: yes □ no □  Location: | | | | |  | | | | | | |
|  | **N195** | | SHO/SpR informed of adverse events | | | |  |  |  | | | | |  |
|  | N196 | | Observations (before treatment) | | | |  |  |  | | | | |  |
|  |  |  | Blood pressure | |  | | | Temperature | | |  | | | |
|  |  |  | Respiration | |  | | | Pulse | | |  | | | |
|  |  |  | Observations (during treatment) | | | |  |  |  | | | | |  |
|  |  |  | Blood pressure | |  | | | Temperature | | |  | | | |
|  |  |  | Respiration | |  | | | Pulse | | |  | | | |
|  |  |  | Observations (after treatment) | | | |  |  |  | | | | |  |
|  |  |  | Blood pressure | |  | | | Temperature | | |  | | | |
|  |  |  | Respiration | |  | | | Pulse | | |  | | | |
| **DATE** | | **TIME** | | Multi-Disciplinary Notes | | | | | | | | **SIGNATURE** | | |
|  | |  | |  | | | | | | | |  | | |
|  | |  | |  | | | | | | | |  | | |
|  | |  | |  | | | | | | | |  | | |
|  | |  | |  | | | | | | | |  | | |
|  | |  | |  | | | | | | | |  | | |
|  | |  | |  | | | | | | | |  | | |
|  | |  | |  | | | | | | | |  | | |
|  | |  | |  | | | | | | | |  | | |
|  | |  | |  | | | | | | | |  | | |
|  | |  | |  | | | | | | | |  | | |
|  | |  | |  | | | | | | | |  | | |
|  | |  | |  | | | | | | | |  | | |
|  | |  | |  | | | | | | | |  | | |
|  | |  | |  | | | | | | | |  | | |
|  | |  | |  | | | | | | | |  | | |
|  | |  | |  | | | | | | | |  | | |
|  | |  | |  | | | | | | | |  | | |

| Day Case - Cut Leishmaniasis ICP | | | | | | AFFIX PATIENT ID LABEL HERE | | | | Date | | |  | |
| --- | --- | --- | --- | --- | --- | --- | --- | --- | --- | --- | --- | --- | --- | --- |
| NURSING | **Code** | | ACTION | | | | Time | **Sign** | **Reason for variance & action taken (use codes)** | | | | | **Sign** |
|  | **Day 27** | | | | | | | | | | | | | |
|  | N197 | | Lesion redressed according to plan | | | |  |  |  | | | | |  |
|  | **N198** | | IV sodium stibogluconate administered (ref. p. 105) | | | |  |  |  | | | | |  |
|  | **N199** | | Patient observed for any adverse events (record below) | | | |  |  |  | | | | |  |
|  | **N200** | | **Adverse Events** | | | | | **Action taken** | | | | | | |
|  |  |  | Nausea: yes □ no □ | | | | |  | | | | | | |
|  |  |  | Malaise: yes □ no □ | | | | |  | | | | | | |
|  |  |  | Abdominal pain: yes □ no □ | | | | |  | | | | | | |
|  |  |  | Myalgia: yes □ no □ | | | | |  | | | | | | |
|  |  |  | Skin rash: yes □ no □  Location: | | | | |  | | | | | | |
|  | **N201** | | SHO/SpR informed of adverse events | | | |  |  |  | | | | |  |
|  | N202 | | Observations (before treatment) | | | |  |  |  | | | | |  |
|  |  |  | Blood pressure | |  | | | Temperature | | |  | | | |
|  |  |  | Respiration | |  | | | Pulse | | |  | | | |
|  |  |  | Observations (during treatment) | | | |  |  |  | | | | |  |
|  |  |  | Blood pressure | |  | | | Temperature | | |  | | | |
|  |  |  | Respiration | |  | | | Pulse | | |  | | | |
|  |  |  | Observations (after treatment) | | | |  |  |  | | | | |  |
|  |  |  | Blood pressure | |  | | | Temperature | | |  | | | |
|  |  |  | Respiration | |  | | | Pulse | | |  | | | |
| **DATE** | | **TIME** | | Multi-Disciplinary Notes | | | | | | | | **SIGNATURE** | | |
|  | |  | |  | | | | | | | |  | | |
|  | |  | |  | | | | | | | |  | | |
|  | |  | |  | | | | | | | |  | | |
|  | |  | |  | | | | | | | |  | | |
|  | |  | |  | | | | | | | |  | | |
|  | |  | |  | | | | | | | |  | | |
|  | |  | |  | | | | | | | |  | | |
|  | |  | |  | | | | | | | |  | | |
|  | |  | |  | | | | | | | |  | | |
|  | |  | |  | | | | | | | |  | | |
|  | |  | |  | | | | | | | |  | | |
|  | |  | |  | | | | | | | |  | | |
|  | |  | |  | | | | | | | |  | | |
|  | |  | |  | | | | | | | |  | | |
|  | |  | |  | | | | | | | |  | | |
|  | |  | |  | | | | | | | |  | | |
|  | |  | |  | | | | | | | |  | | |
|  | |  | |  | | | | | | | |  | | |

| Day Case - Cut Leishmaniasis ICP | | | | AFFIX PATIENT ID LABEL HERE | | | | Date | |  | |
| --- | --- | --- | --- | --- | --- | --- | --- | --- | --- | --- | --- |
| NURSING | **Code** | ACTION | | | Time | **Sign** | **Reason for variance & action taken (use codes)** | | | | **Sign** |
|  | **Day 28** | | | | | | | | | | |
|  | N203 | Lesion reassessment chart completed (P 66) | | |  |  |  | | | |  |
|  | **N204** | Lesion redressed according to reassessment (P 66) | | |  |  |  | | | |  |
|  | N205 | FBC taken and sent | | |  |  |  | | | |  |
|  | N206 | LFT taken and sent | | |  |  |  | | | |  |
|  | N207 | U & E taken and sent | | |  |  |  | | | |  |
|  | N208 | ECG recorded | | |  |  |  | | | |  |
|  | **N209** | IV sodium stibogluconate administered (ref p. 105) | | |  |  |  | | | |  |
|  | **N210** | Patient observed for any adverse events | | |  |  |  | | | |  |
|  | **N211** | **Adverse Events** | | | | **Action taken** | | | | | |
|  |  | Nausea: yes □ no □ | | | |  | | | | | |
|  |  | Malaise: yes □ no □ | | | |  | | | | | |
|  |  | Abdominal pain: yes □ no □ | | | |  | | | | | |
|  |  | Myalgia: yes □ no □ | | | |  | | | | | |
|  |  | Skin rash: yes □ no □  Location: | | | |  | | | | | |
|  | **N212** | Patient reviewed by SHO or SpR | | |  |  |  | | | |  |
|  | **N213** | Observations (before treatment) | | |  |  |  | | | |  |
|  |  | Blood pressure |  | | | Temperature | | |  | | |
|  |  | Respiration |  | | | Pulse | | |  | | |
|  |  | Observations (during treatment) | | |  |  |  | | | |  |
|  |  | Blood pressure |  | | | Temperature | | |  | | |
|  |  | Respiration |  | | | Pulse | | |  | | |
|  |  | Observations (after treatment) | | |  |  |  | | | |  |
|  |  | Blood pressure |  | | | Temperature | | |  | | |
|  |  | Respiration |  | | | Pulse | | |  | | |
|  |  | **Lesion** Ulcer □ Induration □ Scar/Epithelialising □ | | | | | | | | | |

| Day Case - Cut Leishmaniasis ICP | | | | | | AFFIX PATIENT ID LABEL HERE | | | | | | | Date | |  | |  |
| --- | --- | --- | --- | --- | --- | --- | --- | --- | --- | --- | --- | --- | --- | --- | --- | --- | --- |
| NURSING | **Code** | | ACTION | | | | Time | **Sign** | **Reason for variance & action taken (use codes)** | | | | | | | **Sign** |  |
|  | **Day 28 (cont’d)** | | | | | | | | | | | | | | | |  |
|  | N214 | | **Discharge**   - Patient has been reviewed by SHO/SpR - Confirmed clinically fit for discharge - Patient understands s/he is to contact clinic if unwell in the following few days - Patient taught care of lesion - Patient informed how to obtain dressings when required - Outpatients appointment booked - Patient has no complaints - Discharged from electronic system - If hotel stay patient –ensure hotel aware booking now ended | | | |  |  | | | |  | | | |  |  |
| **DATE** | | **TIME** | | Multi-Disciplinary Notes | | | | | | | | | | **SIGNATURE** | | |  |
|  | |  | |  | | | | | | | | | |  | | |  |
|  | |  | |  | | | | | | | | | |  | | |  |
|  | |  | |  | | | | | | | | | |  | | |  |
|  | |  | |  | | | | | | | | | |  | | |  |
|  | |  | |  | | | | | | | | | |  | | |  |
|  | |  | |  | | | | | | | | | |  | | |  |
|  | |  | |  | | | | | | | | | |  | | |  |
|  | |  | |  | | | | | | | | | |  | | |  |
|  | |  | |  | | | | | | | | | |  | | |  |
|  | |  | |  | | | | | | | | | |  | | |  |
|  | |  | |  | | | | | | | | | |  | | |  |
|  | |  | |  | | | | | | | | | |  | | |  |
|  | |  | |  | | | | | | | | | |  | | |  |
|  | |  | |  | | | | | | | | | |  | | |  |
|  | |  | |  | | | | | | | | | |  | | |  |
|  | |  | |  | | | | | | | | | |  | | |  |
|  | |  | |  | | | | | | | | | |  | | |  |
|  | |  | |  | | | | | | | | | |  | | |  |
|  | |  | |  | | | | | | | | | |  | | |  |
|  | |  | |  | | | | | | | | | |  | | |  |
|  | |  | |  | | | | | | | | | |  | | |  |
|  | |  | |  | | | | | | | | | |  | | |  |
|  | |  | |  | | | | | | | | | |  | | |  |
|  | |  | |  | | | | | | | | | |  | | |  |
|  | |  | |  | | | | | | | | | |  | | |  |
|  | |  | |  | | | | | | | | | |  | | |  |
|  | |  | |  | | | | | | | | | |  | | |  |
|  | |  | |  | | | | | | | | | |  | | |  |
|  | |  | |  | | | | | | | | | |  | | |  |
|  | |  | |  | | | | | | | | | |  | | |  |
| 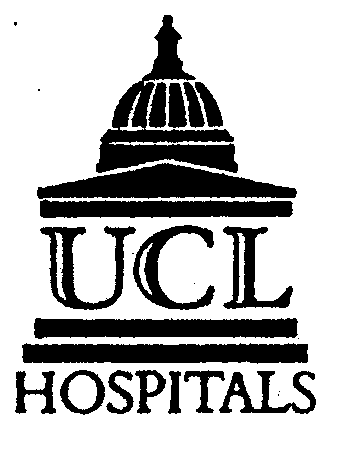  ***Assessment of Lesion***  ***Cutaneous Leishmaniasis***  Date: __________________________________ | | | | | | | | | | Attach Patient Identification Sticker or complete  **PATIENT NAME**  __________________________________________  HOSPITAL NUMBER __________________________________________ | | | | | | | |
| ***LOCATION OF LESION***  ***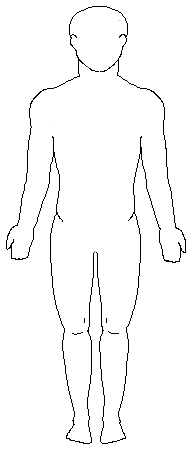*** | | | | | ***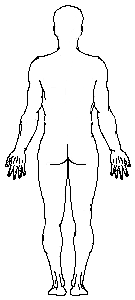*** | | | | | | ***DIAGRAM OF LESION***  (DIMENSIONS IN CM) | | | | | | |

***Initial assessment*** (Nurse to complete)

| **LESION GRADE (From lesion and pressure area care policy) 0 1 2 3 4** | **INFECTION** Swab sent **YES / NO** Date ________ |
| --- | --- |
| **LESION FLOOR CONDITION**  Healthy Granulation **YES / NO**  Thick Slough (Yellow / Brown) **YES / NO** | **RESULT**: Date:________ |
| Necrotic (Black) **YES /NO**  Cellulitis **YES / NO** | **ODOUR YES / NO** |
| **EXUDATE**  Colour  Amount | **PAIN** None  Dressing changes only |
| **CONDITION OF SURROUNDING SKIN**  e.g. Blisters, Fragile, etc. | Continuous  Name of analgesic: |

***Planning*  (Nurse to complete)**

| Debridement Method: |
| --- |
| Cleansing Solution: |
| Topical Agent / Dressing required: |
| Amount of dressing required per dressing change / size of dressing needed (Approx) |
| Frequency of dressing change per day and rationale (e.g. as per protocol) |
| LESION ASSESSED BY (Print & Sign) ________________________ GRADE ___________Review Date __________ |

**PHARMACY DRESSING SUPPLY (Pharmacist to complete)**

| **Dressing** |  |  |  |  |  |
| --- | --- | --- | --- | --- | --- |
| **Quantity supplied** |  |  |  |  |  |
| **Date supplied** |  |  |  |  |  |
| **Signature of Pharmacist** |  |  |  |  |  |

| Day Case - Cut Leishmaniasis ICP | AFFIX PATIENT ID LABEL HERE | Date |  |
| --- | --- | --- | --- |
| - Draw up prescribed 20mg/kg sodium stibogluconate (Pentostam) with a 5 micron filter. - Add drug to 100ml bag of 0.9% normal saline and mix well. - Check site of cannula for phlebitis, signs of infection, potential situation leading to infection, or any other reactions. - Check with patient if there is any experience of pain. - Give 5-10 ml flush of 0.9% normal saline prior to infusion. - Administer drug over 30 minutes. - Check for any adverse reaction or Adverse Events to drug. - Flush off IV infusion line, not just the cannula so that the patient receives the full dose of the medication. - Inform SHOs or SpR if there are any abnormalities in LFT’s/U+E’s and show them the ECG.  Guidance for the administration ofsodium stibogluconate (Pentostam)Reference: | | | |

| Day Case - Cut Leishmaniasis ICP | AFFIX PATIENT ID LABEL HERE | Date |  |
| --- | --- | --- | --- |
| - Admit patient to ward using the Integrated Care Pathways (ICP). - Carry out a nursing assessment.  Treatment  - Cannulate patient. - Take U+E’s and LFT’s (via cannulae if newly inserted or normal venepuncutre if old cannulae) on admission and every 3^rd^ day.   N.B. – Medical staff may decide if appropriate to have PICC lines instead of cannulae. Always check that patients have consented for any procedure and record it.   - ECG on admission and every 3^rd^ day. - Draw up prescribed 10-20mg/kg Sodium Stibogluconate (Pentostam) with a 5 micron filter. - Add drug to 100ml bag of 0.9% normal saline and mix well. - Check site of cannula for phlebitis, signs of infection, potential situation leading to infection, or any other reactions. - Check with patient if there is any experience of pain. - Give 5-10 ml flush of 0.9% normal saline prior to infusion. - Administer drug over 30 minutes (if patient tolerates – can be slowed down as required). - Check for any adverse reaction or side effects to drug. - Flush off IV infusion line, not just the cannula so that the patient receives the full dose of the medication.   Inform SHOs or SpR if there are any abnormalities in LFT’s/U+E’s and show them the ECG. Upper limit of QT interval: 0.420 seconds (420 milliseconds) Nursing Protocolfor Daycase Treatment of Cutaneous Leishmanisis | | | |

| Day Case - Cut Leishmaniasis ICP | AFFIX PATIENT ID LABEL HERE | Date |  |
| --- | --- | --- | --- |
| **Lesion Care**  If there is no reason, such as exudate, to dress the lesion, leave it open. Otherwise:   - Redress the lesion with either a) low-adherent dry dressing e.g. Release (if lesion is clean and non-infected), or b) a hyrdrogel e.g. Intrasite Gel and a foam dressing e.g. allevyn adhesive to deslough lesion. - Reassess lesion site every 7^th^ day (or according to the ICP). - Inform SHOs or SpR if there are any abnormalities in FBC, LFT, and/or U&E - Ensure that SHO or SpR has seen the ECG. - Discharge patient home for the day.  Checks During Treatment Regimen  - At the start of each daycase treatment session, check with patient on Adverse Events and state of well-being when outside clinical area. - Ensure basic observations are taken every day of the treatment regime. - At end of treatment discharge patient from computer.   ~end~ Nursing Protocol (cont’d) | | | |

| Day Case - Cut Leishmaniasis ICP | AFFIX PATIENT ID LABEL HERE | Date |  |
| --- | --- | --- | --- |
| Protocol for Daycase Hotel Stay Patients Patients with Cutaneous Leishmaniasis who fit the criteria for daycase treatment but live outside of the boundaries of the M25 and do not have anywhere/anyone to stay with within the boundaries of the M25 are suitable to be offered hotel accommodation during their daycase treatment on Floor 8.  If the patient fits these above criteria then:   1. Explain to the patient the Trust will pay for B&B hotel accommodation for them whilst   receiving day case treatment at the UCLH instead of being admitted to a hospital bed.  Any extra hotel costs will need to be paid for by the patient and therefore they will need  to provide a credit card or cash deposit to cover any additional costs such as meals &  telephone calls.   1. If the patient can comply with the above please contact either the T8 Ward Sister/ Charge Nurse; Modern Matron; or the General Manager for authorisation to book a hotel place. 2. Once authorisation is given contact the Euston Square Hotel on 020 7388 0099 to make   a reservation. Please speak to either Kaisa; Patricia; Agata; or Luis to make the  reservation. If none of these staff are available the reservation staff will be able to deal  with this.   1. Provide the following details to the reservation team:  - Guest Name   - - Arrival date     - Departure date     - Purchase order number (kept in board holder file) | | | |

| Day Case - Cut Leishmaniasis ICP | AFFIX PATIENT ID LABEL HERE | Date |  |
| --- | --- | --- | --- |
| Protocol for Day case Hotel Stay Patientscontd  1. 5) Once booked please explain to the patient that it is a legal requirement that they must provide the hotel with identification. In addition they should provide the hotel with documentary evidence they are receiving treatment at the UCLH. Therefore please provide the patient with a copy of the day case hotel stay information letter, for which you will need to complete, sign and keep a copy. Please ask the patient to show the hotel this letter when checking in. 2. 6) Provide the patient with a copy of the address, contact details and map to the Euston 3. Square Hotel:   152-156 North Gower St, London, NW1 2LU TEL: +44 (0)20-7388-0099,  FAX: +44-(0)20-7383-7165  [www.euston-square-hotel.com](http://www.euston-square-hotel.com/)   1. Should the patients stay need to be shortened, it is the role of the nurse responsible for day care on that shift to contact the hotel reservations team and inform them that the departure day is being brought forward. The hotel requires at least 24 hours notice in advance of this change to avoid a cancellation fee. This notification of early departure will need to be made in writing either by email to [reservations@euston-square-hotel.com](mailto:reservations@euston-square-hotel.com) or by fax to 020 73837165. Please telephone the reservations team to confirm receipt of this change. 2. Should the patients stay need to be extended please follow the above process from point 2 above to authorise this and extend the booking. 3. On the day of discharge from the day case service please contact the hotel reservations   service (as above) to confirm the patient’s hotel departure date as today. If the patient  has made arrangements to stay longer confirm with the hotel that the UCLH funded stay  is ending as of today. | | | |

**VARIANCE CODES**

| Patient Condition | | | | | | |
| --- | --- | --- | --- | --- | --- | --- |
| 001 | Pyrexia | | 014 | | Lesion infection | |
| 002 | Nausea and/or malaise | | 015 | | Chest infection /UTI | |
| 003 | Pain not controlled | | 016 | | MRSA positive | |
| 004 | Lesion bleeding/oozing | | 017 | | Other infection(Please state in notes) | |
| 005 | Drain oozing/excessive drainage | | 018 | | Poor mobility | |
| 006 | Hypertensive | | 019 | | Mobilising faster than expected | |
| 007 | Hypotensive | | 020 | | Fatigue/sedation | |
| 008 | Pressure area concern | | 021 | | Asleep | |
| 009 | Poor urine output | | 022 | | Confused | |
| 010 | Constipation | | 023 | | Communication problem | |
| 011 | Low blood glucose | | 024 | | Other patient condition issue | |
| 012 | High blood glucose | | 025 | | Deep vein thrombosis | |
| 013 | Catheter in situ | | 026 | | Pulmonary Embolism | |
| Staff / persons | | | | | | |
| 101 | Medical staff decision | 107 | | Families decision | | |
| 102 | Medical staff not available | 108 | | Family unavailable | | |
| 103 | Nursing staff decision | 109 | | Other clinician /PAM decision | | |
| 104 | Nursing staff not available | 110 | | Other clinician /PAM | | |
| 105 | Patients decision | 111 | | Other staff/person issue | | |
| 106 | Patient not available |  | |  | | |
| Departmental system | | | | | | |
| 201 | Done/given previously | | 209 | | | Nursing staff unavailable |
| 202 | Pharmacy delay | | 210 | | | Medical staff unavailable |
| 203 | Trust patient transport delay | | 211 | | | Physiotherapist unavailable |
| 204 | Laboratory delay | | 212 | | | OT unavailable |
| 205 | x-ray delay | | 213 | | | Bed unavailable in unit |
| 206 | Other departmental delay | | 214 | | | Community care unavailable |
| 207 | Equipment unavailable | | 215 | | | Other clinical staff unavailable |
| 208 | Weekend/out of department hours | | 216 | | | Other departmental system issue |
| External System | | | | | | |
| 301 | Bed unavailable in other hospital/unit | | 304 | | | District/ community nurse delay |
| 302 | Other hospital transport delay | | 305 | | | Other community service delay |
| 303 | Social Services delay | | 306 | | | Other external system issue |
| Specific to this ICP | | | | | | |
| 401 | Not registered with a GP | | 407 | | | Results checked the day before |
| 402 | Admitted as inpatient - patient’s choice | | 408 | | | Results not ready |
| 403 | Admitted as inpatient – does not fulfil criteria (please give reasons in MDT notes) | | 409 | | | No adverse events observed or reported |
| 404 | Admitted as inpatient – change of condition (e.g., prolonged QT interval, intolerance of sodium stibogluconate) | | 410 | | | Investigation not required.  Carried out on: [provide date in the relevant column] |
| 405 | Lesion does not need dressing | | 411 | | | Treatment extended |
| 406 | No lesion present | |  | | |  |
